# Exploring prehabilitation interventions for patients with gynaecological cancer undergoing radiotherapy: A scoping review

**DOI:** 10.1371/journal.pone.0319518

**Published:** 2025-03-13

**Authors:** Elizabeth McGladrigan, Elizabeth Wrench, Ewan Dean, Aneurin O’Neil, Lisa Ashmore, Christopher Gaffney

**Affiliations:** 1 Lancaster Medical School, Faculty of Health and Medicine, Lancaster University, Lancaster, Lancashire, United Kingdom; 2 UK Centre for Ecology & Hydrology, Lancaster Environment Centre, Lancaster, Lancashire, United Kingdom; Chinese University of Hong Kong, HONG KONG

## Abstract

**Purpose:**

Radiotherapy imposes a significant physiological and psychological burden on gynaecological cancer patients. Prehabilitation is being increasingly used to prepare individuals for cancer treatment and improve their well-being and resilience. Whilst prehabilitation has demonstrated benefit for individuals undergoing cancer surgery, the structure, role and implementation of prehabilitation prior to radiotherapy are poorly defined and relatively unexplored. This scoping review aims to provide a comprehensive overview of the current literature regarding prehabilitation interventions for individuals with gynaecological cancer undergoing radiotherapy.

**Methods:**

This review was conducted following the gold-standard Joanna Briggs Institute guidelines for scoping reviews. Literature searches were completed in October 2024 across: the Allied and Complementary Medicine Database; British Nursing Index; Cumulative Index to Nursing and Allied Health Literature; Cochrane library (Controlled trials and systematic reviews); Embase; Medical Literature Analysis and Retrieval System Online; and the Psychological Information Database. Grey literature searches were conducted via Google Scholar, Overton.io, and Trip Pro Medical Database.

**Results:**

Ninety records met the inclusion criteria, pertaining to 56 studies. Cervical cancer was the most represented gynaecological cancer type across studies. A small number of multimodal prehabilitation studies were identified (n = 4). Studies evaluating unimodal interventions were more common, with nutritional interventions (n = 24) being the most frequent, followed by psychological (n = 22) and physical exercise (n = 6) interventions. There was considerable variation across studies in respect to intervention initiation, duration, delivery and outcome measures.

**Conclusions:**

The physiological and psychological impacts of cancer diagnosis and treatment are closely entwined. Further development of multimodal prehabilitation to cohesively address these is an important area for future research. Studies evaluating exercise interventions are relatively unexplored in this patient population and the potential barriers to engagement must be considered. Future research should focus on complete and transparent reporting of interventions, with input from those with lived experience, and adopting a standardised set of outcome measures reported across all trials.

## Introduction

Radiotherapy is a mainstay in the treatment of gynaecological malignancies and may be given as a primary treatment, in combination with chemotherapy, and in a neo-adjuvant or adjuvant setting, e.g., for patients receiving surgery [1]. Highly conformal external-beam radiotherapy and brachytherapy techniques have led to reduced morbidity and mortality [2]. However, these treatments still impose a significant physiological and psychosocial burden on patients [1,3].

Radiation-induced cellular death and injury, key to its anti-neoplastic mechanisms, cause adverse effects such as urinary tract and gastrointestinal injury which may present as radiation cystitis, diarrhoea, frequency, urgency, nausea, or bloating [1,2]. Whilst the acute toxicities generally resolve within 12 weeks of completing radiotherapy, many patients experience chronic side-effects that persist months or years later [2,4]. Most radiotherapy side effects are localised to the treatment area, yet fatigue is a commonly reported symptom that may occur acutely or persist long-term [5]. Cancer-related fatigue is characterised by physical, emotional, and/or cognitive tiredness, exhaustion, or weakness related to cancer and its treatment which can significantly alter daily living [5]. Disease and treatment-related burden can also profoundly impact a person’s sexual well-being. Brachytherapy, a technique requiring the insertion of an applicator into the vagina and uterus for prolonged periods, has been highlighted as a source of trauma and distress for gynaecological cancer survivors resulting from factors such as pain, vulnerability, anxiety, and loss of autonomy [6,7]. Unsurprisingly, the acute and chronic side-effects experienced by gynaecological cancer survivors have a profound impact on quality of life, affecting important aspects such as intimacy, social activities, and employment [3,8].

The period between diagnosis and treatment provides an opportunity for early engagement with prehabilitation activities that are designed to optimise patients’ physiological and psychological well-being and resilience [9]. Whilst unimodal exercise regimens have been used by some prior to surgery, prehabilitation is ideally delivered via a multimodal regimen comprising targeted exercise, nutrition and psychological interventions and support [9,10].

Prehabilitation benefits patients undergoing cancer surgery, including reduced complication severity and length of hospital stay [11–13] and prehabilitation interventions for gynaecological cancer patients prior to surgery have been explored in a recent scoping [14] and systematic review [15]. However, the evidence base for prehabilitation is highly variable and there is a comparative dearth of research for prehabilitation prior to non-surgical cancer treatments such as radiotherapy [16]. Additionally, the term prehabilitation is not used ubiquitously in the literature and has only become more frequently used in recent years [9] making it difficult to determine the true extent of the evidence base relating to prehabilitation in this population. Unlike surgery, radiotherapy treatment may be given over a period of weeks providing additional time for patients to participate in health optimising activities. As such, the timing and duration of prehabilitation is less clearly defined for individuals scheduled to undergo radiotherapy treatment. Furthermore, chemotherapy, surgery, radiotherapy, and the various combinations of these treatments all pose unique challenges that necessitate tailored support, meaning prehabilitation developed for surgical cohorts may not be the best approach for those treated with radiotherapy [17]. Therefore, it is of interest to explore prehabilitation in the context of individuals with gynaecological malignancies receiving radiotherapy. In particular, establishing: (1) what prehabilitation interventions are being delivered, (2) their underpinning rationale, (3) the point of delivery and duration of these interventions, and (4) what outcome measures are being reported within the literature. The objective of this scoping review is to provide a comprehensive overview of the current literature regarding prehabilitation interventions for gynaecological cancer patients undergoing radiotherapy.

## Methods

Scoping reviews are a form of evidence synthesis ideal for identifying and mapping the breadth of evidence available for a given topic, clarifying key concepts and highlighting gaps in the literature [18]. They are particularly useful for examining emerging evidence where it is less clear if more precise questions, such as those regarding efficacy, can be asked and suitably addressed in a systematic review [19]. Unlike systematic reviews, the exploratory nature of a scoping review allows for broader research questions and is not intended to assess effectiveness or validity of studies [19].

The methodology for this scoping review was conducted following Joanna Briggs Institute (JBI) guidelines for scoping reviews [20] and reported in accordance with the Preferred Reporting Items for Systematic reviews and Meta-Analyses extension for Scoping Reviews (PRISMA-ScR) checklist [21] (S1 Checklist). The study protocol outlining the objectives, inclusion criteria and methods was registered *a priori* with the Open Science Framework (https://osf.io/jgrv3). This study was a scoping review of the literature and did not require ethical approval.

Preliminary searches of the Cochrane Database of Systematic Reviews, Open Science Framework, JBI Evidence Synthesis and Google were completed in December 2023 and no current or in-progress reviews on this topic for the specified patient population were identified. A subsequent search was conducted in January 2024, following the identification of a recently published scoping review addressing prehabilitation for radiotherapy. However, this review included studies evaluating prehabilitation in any adult patient undergoing radiotherapy, not limited by tumour site, and there were no studies specifically addressing gynaecological cancer. The research questions and inclusion criteria of this proposed review are sufficiently different to those addressed by Flores et al [22] to warrant a further scoping review.

### Inclusion/exclusion criteria

As advised for scoping reviews, the inclusion criteria was developed using the Population, Concept and Context (PCC) framework [20].

We included prospective or retrospective studies that reported on or evaluated (1) prehabilitation for (2) adult (≥18 years old), female patients with a gynaecological malignancy prior to or during radiotherapy, with or without chemotherapy, (3) in any setting where care is provided or an intervention can be delivered to this population. We defined prehabilitation as an intervention prior to or during radiotherapy where unimodal addressed either physical, psychological or nutritional well-being and multimodal delivery was the combination of at least two different categories, e.g., physical and psychological. Preliminary searches indicated there were only a small number of studies evaluating multimodal interventions, as such the authors agreed that inclusion of unimodal prehabilitation interventions was necessary to suitably address the research questions and explore the breadth of prehabilitation literature in this population. Despite prehabilitation sitting within the broader context of health improvement, this review did not include studies focused primarily on smoking cessation, alcohol reduction or medication management, as they as are not encompassed by the primary prehabilitation interventions – tailored physical exercise, nutritional support and psychological support.

Grey literature, conference proceedings, clinical trial protocols and records; and peer-reviewed qualitative, quantitative or mixed-methods publications, were all considered for inclusion. This breadth aligns with a scoping methodology and was considered important to fully gauge the variety of studies evaluating interventions for this population and the various aspects of applying these clinically. Social media posts, animal studies, blogs, and podcasts were excluded. Any relevant systematic or scoping reviews were excluded and hand-searched for primary studies that met the above criteria. Sources were excluded if they were not available in English language due to limited resources available for translation. The authors agreed that studies evaluating prehabilitation interventions in a broader population (e.g., a mixed population of individuals receiving radiotherapy, chemotherapy and/or surgery) would be considered for inclusion if there was sufficient separation in the results for the population of interest, in addition to ongoing clinical trials, as they could provide valuable insight into the review questions.

### Search strategy

An initial search was conducted in Medical Literature Analysis and Retrieval System Online (MEDLINE) and Cumulative Index to Nursing and Allied Health Literature (CINAHL) with an adapted version of the terms used by Saggu *et al* [14]. The full search strategy was then developed using an analysis of text words contained within the titles and abstracts of retrieved papers and any index terms used to describe the articles. This search strategy was adapted and applied to each database including: Allied and Complementary Medicine Database (AMED) (Ovid and ProQuest Dialog); British Nursing Index (BNI) (ProQuest); CINAHL (EBSCO); Cochrane library (Controlled trials and systematic reviews); Embase (Ovid); MEDLINE (EBSCO); Psychological Information Database (PsycINFO) (EBSCO). Each search strategy was then peer reviewed by a research librarian and modified according to feedback. A search for grey literature was also conducted via Google Scholar, Overton.io, and Trip Pro Medical Database. Final searches were completed in October 2024. The full search strategies are available in the supplementary materials (S1 Table and S2 Table). Included sources and identified reviews were hand-searched for additional eligible articles.

### Study selection

All identified records were uploaded to EndNote (Clarivate Analytics, PA, USA) and duplicates removed. The remaining abstracts were then uploaded to Rayyan (Qatar Computing Research Institute, Doha, Qatar) for title and abstract screening by the primary reviewer (EM) and the secondary screening was divided equally between two independent reviewers (ED and EW). The full-text screening for any potentially relevant sources were then screened in duplicate by the reviewers (EM, EW, ED or AON). Any conflicts were resolved by discussion between the relevant reviewers. A small number of sources (n = 8) were unable to be uploaded to Rayyan, the title and abstracts of each source were reviewed against the inclusion criteria and were found not to be relevant to the review.

### Data charting

Relevant data were charted using a standardised form developed for this study by a reviewer (EM). This data extraction form was developed in accordance with JBI guidance [20] and piloted across different sources by two reviewers (EM and EW). Where multiple sources for the same studies were identified, the records were collated for data extraction to minimise risk of double counting [23]. A random sample (n = 8) were then verified by independent reviewers (ED or AON). Data items were extracted as published, including: title, author, year of publication, aims/purpose, population, study design, intervention type, comparator; and outcome measures, along with items from the Template for Intervention Description and Replication (TIDieR) checklist [24]. In the case of ongoing trials, all data items were extracted from available clinical trial records or published study protocols including planned intervention components and outcome measures. Authors were contacted for missing or additional information with a subsequent follow-up email after a minimum of two weeks, as necessary. Critical appraisal of the quality of included studies was not performed as this is not generally recommended for scoping reviews [19,21]. Data are presented in tabular and diagrammatic formats accompanied by a descriptive summary in a manner that aligns with the review objectives. Figures were prepared in GraphPad Prism 10 (GraphPad Software 2365 Northside Dr. Suite 560 San Diego, CA 92108) and the GNU Image Manipulation Programme (GIMP 2.10.38, gimp.org), unless otherwise stated.

## Results

### Study selection and descriptive characteristics of the studies

**Fig 1:**
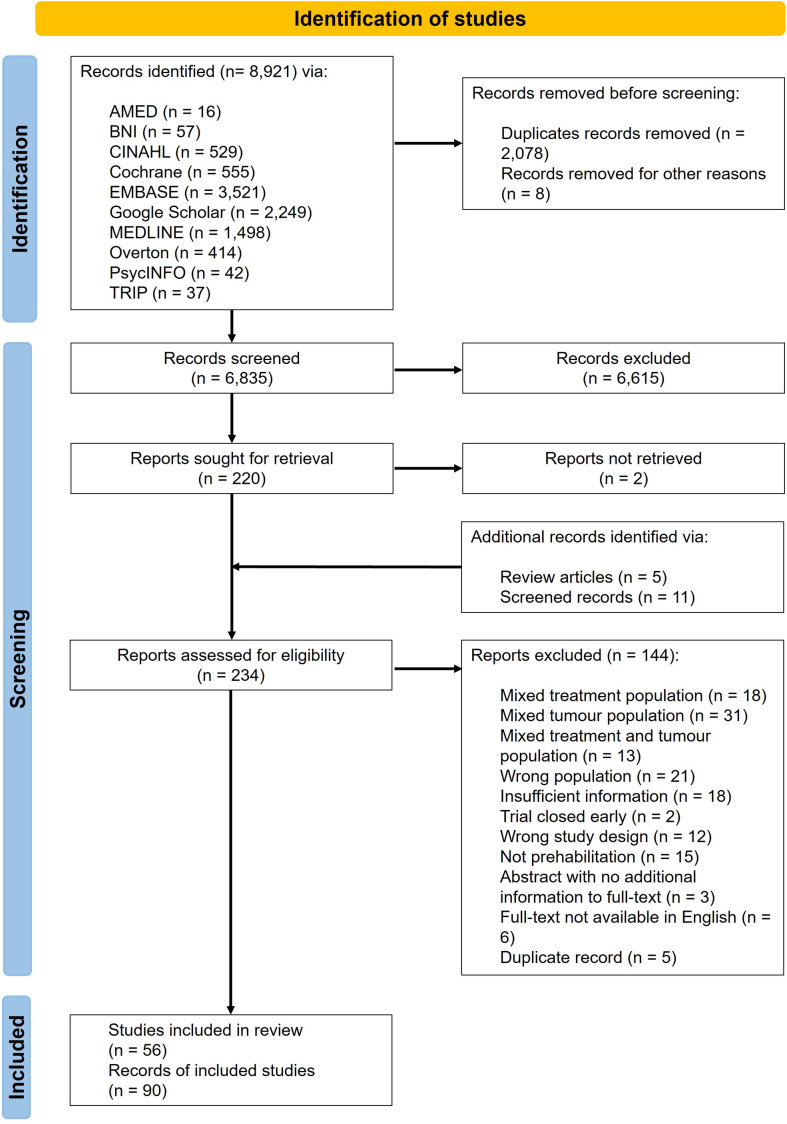
PRISMA flow diagram of study selection process.

The systematic search identified 8,921 records (Fig 1). Following deduplication in EndNote, 6,835 titles and abstracts were screened against the inclusion criteria. A total of 234 records were then further assessed for eligibility, 90 were included pertaining to 56 studies.

**Fig 2:**
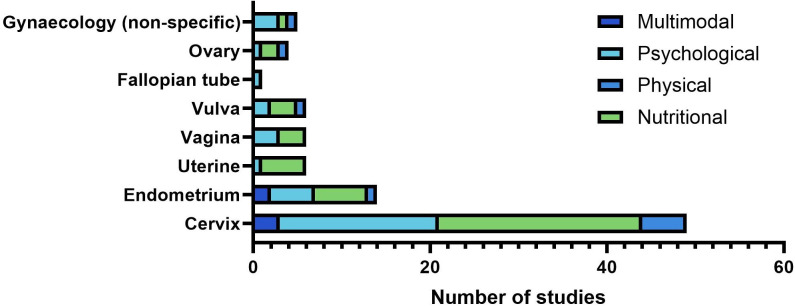
Number of studies including different gynaecological cancers by type.

The characteristics and details of the included studies are summarised in Tables 1–6. The 56 studies were categorised as multimodal (n = 4) [25–33], physical/exercise (n = 6) [34–42], psychological (n = 22) [43–79], and nutritional (n = 24) [80–114]. The studies were conducted in Australia (n = 4), Brazil (n = 2), Canada (n = 2), Chile (n = 1), China (n = 5), England (n = 1), France (n = 2), Finland (n = 1), Germany (n = 2), Italy (n = 1), India (n = 8), Japan (n = 1), Republic of Korea (n = 1), Mexico (n = 4), Myanmar (n = 1), Norway (n = 2), Spain (n = 5), Sweden (n = 2), Thailand (n = 2), Turkey (n = 1), and the USA (n = 8). Several ongoing clinical trials were included (n = 10) [28,36,39,43,51,72,73,91,102,108] and the earliest included record was published in 1958 [104]. Sources included peer-reviewed journal articles (n = 50), conference abstracts (n = 13), clinical trial records (n = 20), peer-reviewed study protocols (n = 4), a pre-print author manuscript (n = 1) and dissertations (n = 2). Cervical cancer was the most highly represented gynaecological cancer type across study populations, however, studies also included endometrial, uterine, vaginal, vulval, ovarian and fallopian tube malignancies (Fig 2).

**Table 1:** Characteristics of included completed studies.

Title (clinical trial identifier)	Author and year	Source type	Country	Aims/purpose	Population	Study design
Multimodal						
ENhAncing Lifestyle Behaviors in EndometriaL CancEr (ENABLE): A Pilot Randomized Controlled Trial (ACTRN12619000631101)	Edbrooke et al (2019, 2020, 2022) [25,29,30]	Clinical trial record (2019) Conference abstract (2020) Peer-reviewed journal article (2022)	Australia	To assess the feasibility and safety of an allied health intervention during adjuvant treatment	Patients with endometrial cancer scheduled for adjuvant treatment (BT, external beam XRT or concurrent CXRT followed by adjuvant CTx) following surgery	Pilot RCT
Enhanced Recovery Pathway (ERP) in Patients Undergoing Brachytherapy for Gynecologic Cancer (GYN-BT)	Andring et al (2022a, 2022b, 2023) [26,31,32]	Conference abstracts	USA	To implement an ERP for patients with gynaecological malignancies, scheduled for BT, to standardise and optimise the peri-operative phase and evaluate the impact on patient experience	Patients with cervical cancer scheduled for BT	Quasi-experimental with a non-equivalent control group
Prehabilitation in Locally Advanced Cervical Cancer Patients Receiving Radiotherapy	Kaliamurthi et al (2022) [27]	Conference abstract	India	To assess the impact of prehabilitation on cervical cancer patients receiving XRT ± CTx on OTT, HRQoL and to assess the treatment related morbidity requiring hospital admission	Patients with locally advanced cervical cancer scheduled for XRT ± CTx followed by BT	Single-arm interventional study (pre-post study)
Physical/exercise						
Strengthening of Pelvic Floor Muscles for Incontinence in Cervical Cancer	Jagdish et al (2022) [38] Jagdish and Daptardar (2023) [42]	Conference abstract (2022) Pre-print author manuscript (2023)	India	To analyse the effect of PFME on urinary incontinence in patients with cervical cancer	Patients with cervical cancer scheduled to undergo external beam XRT and BT	Single-arm interventional study (pre-post study)
Pre-rehabilitation of the pelvic floor before radiation therapy for cervical cancer: a pilot study	Sacomori et al (2020) [35] Araya-Castro et al (2020) [40]	Peer-reviewed journal articles	Chile	To evaluate the influence of teaching PFMEs prior to external beam XRT or BT on pelvic floor muscle function	Patients with cervical cancer referred for curative XRT and/or BT	Pilot single-arm interventional study (pre-post study)
Prophylactic complex physiotherapy in gynecologic cancer survivors: patient-reported outcomes based on a lymphedema questionnaire	Daggez et al (2023) [34]	Peer-reviewed journal article	Turkey	To evaluate prophylactic complex physiotherapy in gynaecological cancer patients and the effect on patient-reported symptoms	Patients with gynaecological (Endometrial, ovarian, cervical, vulvar) cancer who underwent a lymphadenectomy and were recommended for prophylactic complex physiotherapy prior to adjuvant treatment (CTx, XRT, CXRT)	Cohort study
Exercise during treatment for advanced cervical cancer	Tórtola-Navarro (2023) [37]	Peer-reviewed journal article	Spain	To design an individualised physical activity (PA) programme to be delivered during treatment to test its feasibility and evaluate the impact on the patient’s side effects, functional status and QoL	A patient with cervical cancer scheduled for CXRT and posterior BT	Case study
Psychological						
A nurse led psychosocial intervention with peer support to reduce psychosocial needs in women with gynaecological cancer (ACTRN12609000312246)	Schofield (2009) [46] Bergin et al (2016) [69]	Clinical trial record (2009) Peer-reviewed journal article (2016)	Australia	To develop a nurse-led psychosocial intervention with peer support for patients with gynaecological cancers to address their psychosocial and psychosexual needs; reduce anxiety and symptom distress	Patients with gynaecological cancer scheduled for curative XRT	Pilot single-arm study
PeNTAGOn: A nurse- and peer-led psycho-educational intervention to support women with gynaecological cancers receiving curative radiotherapy (ACTRN12611000744954)	Schofield et al (2011, 2013, 2020) [48,49,68] Gough et al (2022) [47]	Clinical trial record (2011) Peer-reviewed study protocol (2013) Peer-reviewed journal articles (2020, 2022)	Australia	To evaluate the impact of a psychoeducational intervention at reducing distress in patients with gynaecological cancers receiving curative XRT	Patients with cervical, endometrial/uterine, vaginal, vulvar, ovarian, fallopian tube or other gynaecological cancers scheduled for external beam XRT, BT ± CTx, CXRT or a combination	RCT
A study to assess effectiveness of social support group among cervical cancer patients and their caregivers (CTRI/2019/07/020057)	Thakur (2019) [78] Thakur et al (2021) [61]	Clinical trial record (2019) Peer-reviewed journal article (2021)	India	To evaluate the effectiveness of a support group intervention on the levels of pain and fatigue experienced by cervical cancer patients	Patients with cervical cancer receiving CXRT	Quasi-experimental study with total enumeration sampling
A Randomized Controlled Trial on Pranayama and Yoga Nidra for Anxiety and Depression in Patients With Cervical Cancer Undergoing Standard of Care (CTRI/2022/02/040423)	Patil (2022) [77] Nuzhath et al (2024) [59]	Clinical trial record (2022) Peer-reviewed journal article (2024)	India	To evaluate the effect of pranayama and yoga nidra on levels of anxiety and depression in cervical cancer patients undergoing CXRT and BT	Patients with cervical cancer scheduled for CXRT and BT	RCT
A randomized trial of the effect of training in relaxation and guided imagery techniques in improving psychological and quality-of-life indices for gynecologic and breast brachytherapy patients	León-Pizarro et al (2007) [60]	Peer-reviewed journal article	Spain	To determine the efficacy of a relaxation intervention including guided imagery at reducing anxiety and depression in patients with gynaecological or breast cancer receiving BT	Patients with breast or gynaecological cancer scheduled for BT	RCT
An effective group psychoeducational intervention for improving compliance with vaginal dilation: A randomized controlled trial	Jeffries (2002) [44] Jeffries et al (2006) [52]	Dissertation (2002) Peer-reviewed journal article (2006)	Canada	To evaluate the impact of a group psychoeducational sexual intervention on sexual health including compliance with vaginal dilation	Patients with cervical or endometrial cancer scheduled for XRT	RCT
Assessment of an onco-sexology support and follow-up program in cervical or vaginal cancer patients undergoing brachytherapy	Kpoghomou et al (2021) [70]	Peer-reviewed journal article	France	To evaluate the impact of an onco-sexology support and follow-up intervention on sexuality, late-effects and clinical monitoring	Patients with cervical or vaginal cancer scheduled for external beam XRT and BT	Cohort study
Effect of Foot Reflexology and Aromatherapy on Anxiety and Pain During Brachytherapy for Cervical Cancer	Blackburn et al (2021) [62]	Peer-reviewed journal article	USA	To determine if aromatherapy and reflexology impact levels of pain and anxiety during BT	Patients with cervical cancer scheduled for BT	RCT
Effect of guided imagery relaxation on anxiety in cervical cancer: randomized clinical trial (RBR-7ssvytb)	de Oliveira Santana et al (2021, 2023) [45,67]	Clinical trial record (2021) Peer-reviewed journal article (2023)	Brazil	To evaluate the effect of virtual reality image guided relaxation	Patients with cervical cancer scheduled for CXRT	RCT
Effectiveness of an Interventional Package on the Level of Anxiety, Depression, and Fatigue among Patients with Cervical Cancer (CTRI/2017/06/008732)	Kaur (2017) [79] Kaur et al (2018) [65]	Clinical trial record (2017) Peer-reviewed journal article (2018)	India	To evaluate the effect of an interventional package including progressive muscle relaxation, counselling and self-care techniques on levels of anxiety, depression and fatigue	Patients with cervical cancer scheduled for CXRT	Quasi-experimental study with total enumeration sampling
Effectiveness of Yoga Nidra in Mitigating Stress in Women Undergoing Curative Radiotherapy for Cervical Cancer	D’cunha et al (2021) [64]	Peer-reviewed journal article	India	To evaluate the effect of structured relaxation exercise through yoga nidra on stress levels	Patients with cervical cancer scheduled to receive CXRT	RCT
Effects of Integrated Music-Video Therapy on Pain and Anxiety During High-Dose-Rate Brachytherapy	Lim (2024) [71]	Peer-reviewed journal article	Republic of Korea	To examine the effect of an integrated music-video therapy on pain and anxiety levels experienced by individuals with gynaecological cancer at different stages of BT	Patients with cervical cancer receiving XRT ( ± CTx) and BT	Single group crossover study
Effects of mindfulness-based stress reduction on cervical cancer patients undergoing concurrent radiochemotherapy	An et al (2020) [63]	Peer-reviewed journal article	China	To evaluate the effect of mindfulness based stress reduction on side effects, self-perceived burden and QoL	Patients with cervical cancer scheduled for CXRT	Cohort study
Humanity Assurance Protocol in Interventional RadiotheraPY (HAPPY)	Lancellotta et al (2019, 2023) [50,76] Tagliaferri et al (2024) [58]	Peer-reviewed journal articles (2019, 2024) Conference abstract (2023)	Italy	To propose a series of suggestions and interventions to enhance patients’ psychological well-being and evaluate their effectiveness when implemented	Patients with endometrial or cervical cancer scheduled for BT following surgery ± external beam XRT	Single-arm interventional study (pre-post study)
Impact of early institution of Palliative care on Quality of Life of patients with locally advanced cancer of the uterine cervix- A prospective randomized study. - EIPAQ-CX (CTRI/2017/05/008704)	Rai (2017) [75] Dey et al (2023) [57]	Clinical trial record (2017) Peer-reviewed journal article (2023)	India	To evaluate the feasibility and impact of an early palliative care intervention on QoL for cervical cancer patients undergoing radical CXRT	Patients with cervical cancer scheduled for radical CXRT and BT	Pilot RCT
Music Relaxation Video and Pain Control: A Randomized Controlled Trial For Women Receiving Intracavitary Brachytherapy For Gynaecological Cancer	Chi et al (2011, 2015) [56,74] Chi (2009) [66]	Conference abstract (2011) Dissertation (2009) Peer-reviewed journal article (2015)	USA	To evaluate the effects of music relaxation videos on pain, opioid use and anxiety in patients scheduled for BT	Patients with cervical cancer scheduled for BT	RCT
Preservation of Immune Function in Cervical Cancer Patients during Chemoradiation using a Novel Integrative Approach	Lutgendorf et al (2010) [54] Hart et al (2011) [55]	Peer-reviewed journal articles	USA	To evaluate the effects of healing touch therapy or relaxation training on immune cell activity, mood and QoL	Patients with cervical cancer scheduled for CXRT and BT	RCT
Regular counselling by an oncology nurse increases coping with side effects during outpatients radiotherapy of gynecological malignancies	Varre et al (1999) [53]	Conference abstract	Norway	To evaluate the effect of nurse administered counselling on anxiety, coping and QoL	Patients with gynaecological cancer scheduled for XRT	Pilot RCT
Nutritional						
A phase 2 randomized controlled trial of oral resistant starch supplements in the prevention of acute radiation proctitis in patients treated for cervical cancer	Sasidharan et al (2016, 2019) [98,99]	Conference abstract (2016) Peer-reviewed journal article (2019)	India	To evaluate the benefits of oral prebiotic amylase resistant starch in reducing the incidence of radiation proctitis in cervical cancer patients	Patients with cervical cancer scheduled for radical CXRT	Double-blind, RCT with placebo
A Randomized, Double-Blind Pilot Trial of Hydrolyzed Rice Bran versus Placebo for Radioprotective Effect on Acute Gastroenteritis Secondary to Chemoradiotherapy in Patients with Cervical Cancer (UMIN000004350)	Itoh et al (2015) [90]	Peer-reviewed journal article	Japan	To evaluate the radioprotective effect of hydrolysed rice bran on the severity of CXRT related acute gastroenteritis in cervical cancer patients	Patients with cervical cancer scheduled for CXRT ± BT	Double-blind, pilot RCT with placebo
Arginine, glutamine, and fish oil supplementation in cancer patients treated with concurrent chemoradiotherapy: A randomized control study	Chitapanarux et al (2020) [83]	Peer-reviewed journal article	Thailand	To evaluate the effect of arginine, glutamine and fish oil supplements on CXRT completion rates and toxicities	Patients with cervical, oesophageal or head and neck cancer scheduled for definitive or adjuvant CXRT	RCT
Changes of immune response and side effects before and after nutritional intervention in cervical cancer patients with concurrent chemoradiotherapy	Chen (2018) [114]	Peer-reviewed journal article	China	To investigate the impact of a targeted nutritional intervention on immune response and side effects in cervical cancer patients	Patients with cervical cancer scheduled for CXRT	Cohort study
Decreasing the Adverse Effects in Pelvic Radiation Therapy: A Randomized Controlled Trial Evaluating the Use of Probiotics (NCT02351089)	Ahrén et al (2022) [81]	Peer-reviewed journal article	Sweden	To evaluate the benefit of probiotics XRT-related GI side effects	Patients with cervical, corpus uteri, vaginal or vulval cancer scheduled for primary or adjuvant external beam XRT ± CTx	Double-blind, RCT with placebo
Dietary regime during radiation therapy for carcinoma of the uterus	Turner (1958) [104]	Peer-reviewed journal article	Australia	To describe the impact of a dietary regime on side effects and patient well-being	Patients with cervical cancer receiving external beam XRT and BT	Single-arm interventional (pre-post) study
Effect of an Anti-inflammatory Diet on Patients with Cervical Cancer (NCT03994055)	Cetina (2019) [112] J. Luvián-Morales et al (2023) [94]	Clinical trial record (2019) Conference abstract (2023)	Mexico	To compare the effects of an anti-inflammatory diet and a low residue diet on nutritional status and GI toxicity	Patients with cervical cancer scheduled for CXRT and BT	RCT
Effect of Probiotics for the Prevention of Acute Radiation-Induced Diarrhoea Among Cervical Cancer Patients: a Randomized Double-Blind Placebo-Controlled Study (TCTR20170314001)	Linn et al (2019) [93]	Peer-reviewed journal article	Myanmar	To investigate the impact of a probiotic supplement in prevention of radiation-induced diarrhoea	Patients with cervical cancer scheduled for CXRT	Double-blind, RCT with placebo
Effectiveness of a nutritional intervention in the reduction of gastrointestinal toxicity during teletherapy in women with gynaecological tumours	Soto-Lugo et al (2017, 2018) [100,101]	Peer-reviewed journal articles	Mexico	To evaluate whether a dietary intervention low in oligosaccharides, disaccharides, monosaccharides and fermentable polyols reduces XRT-related acute GI side effects	Patients with cervical or endometrial cancer scheduled for radical or adjuvant external beam XRT ± CTx	RCT
Efficacy of ω-3 supplementation on nutritional status, skeletal muscle, and chemoradiotherapy toxicity in cervical cancer patients (NCT02779868)	Chaves (2016) [113] Aredes et al (2017, 2019) [92,103]	Clinical trial record (2016) Conference abstract (2017) Peer-reviewed journal article (2019)	Brazil	To evaluate the effect of ω-3 supplementation on body composition in cervical cancer patients receiving CXRT	Patients with cervical cancer undergoing CXRT who are at nutritional risk or are experiencing malnutrition	Triple-blind, RCT with placebo
Effect of inulin and fructo-oligosaccharide on the prevention of acute radiation enteritis in patients with gynecological cancer and impact on quality-of-life: a randomized, double-blind, placebo-controlled trial (NCT01549782)	García-Peris et al (2012, 2016) [86,87]	Peer-reviewed journal articles	Spain	To evaluate the effect of a fibre mixture containing inulin and fructo-oligosaccharide on GI toxicity, QoL and the effects of pelvic radiotherapy on intestinal microbiota	Patients with endometrial, cervical, uterine, vulval or vaginal cancer scheduled for external beam XRT and BT	Double-blind, RCT with placebo
Effects of Probiotic *Lactobacillus Casei* DN-114 001 in Prevention of Radiation-Induced Diarrhea: Results From Multicenter, Randomized, Placebo-Controlled Nutritional Trial	Giralt et al (2008) [88]	Peer-reviewed journal article	Spain	To evaluate the impact of a probiotic drink on the incidence of XRT-induced diarrhoea	Patients with endometrial cancer scheduled for adjuvant XRT or cervical cancer patients scheduled for CXRT	Double-blind, RCT with placebo
Efficacy of Glutamine in the Prevention of Acute Radiation Enteritis (NCT00828399)	Vidal-Casariego et al (2014) [105]	Peer-reviewed journal article	Spain	To assess if glutamine has a proactive effect for patients receiving pelvic XRT to reduce radiation enteritis	Patients with endometrial, cervical or other pelvic/abdominal malignancies scheduled for XRT	Double-blind, RCT with placebo
Effect of symbiotic supplementation on fecal calprotectin levels and lactic acid bacteria, Bifidobacteria, Escherichia coli and Salmonella DNA in patients with cervical cancer	de Loera-Rodriguez et al (2018) [85]	Peer-reviewed journal article	Mexico	To evaluate the impact of synbiotic supplements on faecal calprotectin levels, bacterial DNA levels and GI side effects	Patients with cervical cancer scheduled for CXRT	Double-blind, RCT with placebo
Multicenter, Phase 3 Trial Comparing Selenium Supplementation With Observation in Gynecologic Radiation Oncology	Muecke et al (2010) [96]	Peer-reviewed journal article	Germany	To assess the impact of oral selenium supplementation on selenium levels and XRT side effects	Patients with cervical or corpus uteri cancer scheduled for adjuvant external beam XRT ± BT	RCT
Phase II Study Assessing the Feasibility of Using Elemental Supplements to Reduce Acute Enteritis in Patients Receiving Radical Pelvic Radiotherapy	Craighead and Young (1998) [84]	Peer-reviewed journal article	Canada	To assess patients’ compliance with an elemental dietary supplement regime and its efficacy in preventing acute radiation enteritis	Patients with endometrial or cervical cancer scheduled for adjuvant or radical external beam XRT ± BT	Quasi-experimental study with non-equivalent control group
Preservation of intestinal integrity during radiotherapy using live *Lactobacillus acidophilus* cultures	Salminen et al (1988) [97]	Peer-reviewed journal article	Finland	To evaluate the impact of a dietary intervention including *Lactobacillus acidophilus* cultures on GI toxicities	Patients with cervix or uterine cancer scheduled for external beam XRT and BT	RCT
Randomized controlled trial of live lactobacillus acidophilus plus bifidobacterium bifidum in prophylaxis of diarrhea during radiotherapy in cervical cancer patients	Chitapanarux et al (2010) [82]	Peer-reviewed journal article	Thailand	To evaluate the effect of an oral probiotic containing live *lactobacillus acidophilus* and *bifidobacterium bifidum* on incidence and severity of XRT-induced diarrhoea	Patients with cervical cancer external beam CXRT and BT	Double-blind, RCT with placebo
Repurposing Individualized Nutritional Intervention as a Therapeutic Component to Prevent the Adverse Effects of Radiotherapy in Patients With Cervical Cancer	Medina-Jiménez and Monroy-Torres (2020) [95]	Peer-reviewed journal article	Mexico	To evaluate the impact of a tailored nutritional intervention with counselling on weight change and GI toxicities	Patients with cervical cancer scheduled for XRT	Quasi-experimental study with a retrospective comparison group
The effect of selenium supplementation on the efficacy of concurrent radiotherapy for cervical cancer: a randomized, double-blind, placebo-controlled phase II clinical trial (ChiCTR2100043379)	Huang (2021) [89] Yang et al (2023) [106]	Clinical trial record (2021) Peer-reviewed journal article (2023)	China	To evaluate the safety and efficacy of selenium supplementation in reducing CXRT haematological toxicity	Patients with cervical cancer scheduled for primary CXRT and BT	Double-blind, RCT with placebo
The effect of a low fat, low lactose diet during pelvic radiotherapy	Bye et al (1992, 1993, 1995) [109–111]	Peer-reviewed journal articles	Norway	To evaluate the impact of a low fat, low lactose dietary intervention on nutritional status, GI toxicities and QoL	Patients with endometrial, ovarian or cervical cancer external beam XRT ± BT	RCT

*Abbreviations*: BT =  brachytherapy, CTx =  chemotherapy, CXRT =  chemoradiotherapy, ERP =  enhanced recover pathway, GI =  gastrointestinal, HRQoL =  health related quality of life, OTT =  overall treatment time, PFME =  pelvic floor muscle strengthening exercises, QoL =  Quality of life, RCT =  randomised controlled trial, XRT =  radiotherapy.

**Table 2:** Characteristics of included ongoing clinical trials.

Title (clinical trial identifier)	Author and year	Source type	Country	Aims/purpose	Population	Study design
Multimodal						
RadBone: bone toxicity following pelvic radiotherapy (NCT04555317)	The Christie NHS Foundation Trust (Sponsor) (2020) [33] Grigoriadou et al (2022) [28]	Clinical trial record (2020) Peer-reviewed study protocol (2022)	England	To determine the feasibility and acceptability of a musculoskeletal health package intervention and remote or in-person prehabilitation in women undergoing radiotherapy for gynaecological malignancies	Patients with endometrial or cervical cancer undergoing curative or adjuvant radiotherapy	RCT
Physical/exercise						
Early Intervention to Prevent Lower Limb Lymphedema of Gynecological Malignancy (NCT05793749)	Zou (2023)[39]	Clinical trial record	China	To evaluate the impact of prophylactic physiotherapy on the incidence of lymphoedema and QoL in patients with gynaecological cancers	Patients with gynaecological malignancies scheduled for adjuvant XRT following lymphadenectomy	RCT
Randomised Trial Evaluating the Benefit of a Fitness Tracker Based Workout During Radiotherapy (OnkoFit II) (NCT04517019)	Gani (2020) [41] Hauth et al (2021) [36]	Clinical trial record (2020) Peer-reviewed study protocol (2021)	Germany	To investigate the impact of an activity tracker-based fitness programme on cancer-related fatigue, QoL and pre-operative health status of patients undergoing radiotherapy	Patients with cervix uteri, lung, brain, head and neck, pancreatic, rectal or oesophageal cancer or sarcoma indicated for preoperative, definitive or postoperative CXRT	RCT
Psychological						
Impact of an Educational Physiotherapy-Yoga Intervention on Perceived Stress in Women Treated With Brachytherapy for Cervical Cancer (KYOCOL) (NCT06263283)	Texier and Meignant (2024) [72]	Clinical trial record	France	To evaluate the impact of an educational physiotherapy-yoga intervention on perceived stress	Individuals with cervical cancer scheduled for BT with a stress level ≥ 3 (Visual analogue scale 0 to 10)	RCT
Study Protocol for the Social Interventions for Support During Treatment for Endometrial Cancer and Recurrence (SISTER) study: a community engaged national randomized trial	Oluloro et al (2024) [73]	Peer-reviewed study protocol	USA	To determine whether virtual support interventions improve treatment completion, compare the efficacy of these interventions on the level of social isolation during cancer treatment amongst Black women with high-risk endometrial cancer, and evaluate the barriers and facilitators to social support delivery for patients, providers and cancer centre leaders	Black/African American individuals with high-risk endometrial cancer scheduled for adjuvant CTx, XRT or immunotherapy	RCT
Using Reiki Therapy to Improve Symptoms Associated With Brachytherapy in Patients With Gynecological Malignancies (Reiki-Brachy) (NCT05979610)	Burt (2023) [43]	Clinical trial record	USA	To evaluate the impact of reiki therapy on pain and anxiety in patients receiving BT	Patients with cervical, endometrial, vaginal or vulval cancer scheduled for BT	RCT
Yoga Therapy During Chemotherapy and Radiation Treatment for the Improvement of Physical and Emotional Well-Being in Patients With Stage IB2-IIIB Cervical Cancer (NCT04622670)	Ramondetta (2020) [51]	Clinical trial record	USA	To establish the feasibility of delivering a yoga intervention during CXRT and evaluate the effect of a yoga intervention during CXRT on physical and emotional well-being, including fatigue, depression, anxiety, pain and QOL	Patients with cervical cancer scheduled for curative CXRT	RCT
Nutritional						
Dietary Fiber During Radiotherapy and Intestinal Inflammation - a Placebo-controlled Randomized Trial (FIDURA) (NCT04534075)	Steineck (2020) [102] Ahlin et al (2021) [80]	Clinical trial record (2020) Peer-reviewed journal article (2021)	Sweden	To investigate whether additional dietary fibre has benefits on long-term intestinal health for cancer survivors	Patients with a pelvic cavity tumour (including gynaecological cancer) scheduled for neoadjuvant or curative XRT	Triple-blind, RCT with placebo
The Effects of Immunonutrition Therapy on the Nutritional Status, Immune Function, and Quality of Life of Locally Advanced Cervical Cancer Patients With Malnutrition: an Open-label Randomized Controlled Study (NCT06349148)	Shuang-Zheng (2024) [108]	Clinical trial record	China	To evaluate the effect of immunonutrition on individuals with cervical cancer treated with concurrent CXRT	Individuals with locally advanced cervical cancer undergoing XRT ± CTx with moderate or severe malnutrition	RCT
Time-Restricted Eating Versus Nutritional Counseling for the Reduction of Radiation or Chemoradiation Tx Side Effects in Patients With Prostate, Cervical, or Rectal Cancers (NCT05722288)	Li (2023) [91] Eustace et al (2024) [107]	Clinical trial record (2023) Conference abstract (2024)	USA	To evaluate the effect of time-restricted eating on CXRT side effects compared to nutritional counselling	Patients with cervical or rectal cancer scheduled for CXRT or prostate cancer scheduled for XRT ^+^ androgen deprivation therapy	RCT

**Table 3:** Overview of the studies evaluating multimodal prehabilitation interventions using a modified TIDieR checklist.

Study	Why	What	Who	When and how much
ENhAncing Lifestyle Behaviors in EndometriaL CancEr (ENABLE) [25,29,30]	Patients have reported high levels of distress and support needs. Endometrial cancer patients have expressed a desire for additional support and monitoring as an external motivation to successfully change health behaviours. A low-cost allied health intervention has potential to improve outcomes. Positive association reported between meeting physical activity guidelines and HRQoL Behaviour change strategies were based on HealthChange® methodologies Intervention timing close to diagnosis during adjuvant treatment to provide active coping strategies and patients may be more open to making lifestyle changes	Participants in the intervention group received a multi-disciplinary program of social support, lifestyle education, and behavioural change support. The program included 1:1 sessions on diet, PA, and weight management, starting with a face-to-face session and followed by remote sessions Individualised education was provided including home-based PA programs, patient-centred nutrition goals, and behaviour change strategies. Optional tools included a pedometer, diary, and motivational text messages Social work intervention was tailored according to the individual’s needs and support service referrals were made as necessary Participants were provided with the Cancer Council’s educational booklet “Exercise for People Living with Cancer” Usual care participants received routine support, with referrals to social work or psychology if indicated but no structured weight management or exercise support	Dietician, physiotherapist and social worker (with 5, 6, and 13 years of oncology rehabilitation experience respectively)	Pre-, during and post adjuvant treatment - initiation variable as patients may be having BT, XRT or CXRT 8 weeks Initial multi-disciplinary meeting (approx. 1.5 hours) with weekly video or telephone follow-ups (20-30 minutes) Recommended that participants engage in moderate intensity exercise for ≥ 10 minutes, working towards a goal of 150 minutes of aerobic exercise per week
Enhanced Recovery Pathway (ERP) in Patients Undergoing Brachytherapy for Gynecologic Cancer (GYN-BT) [26,31,32]	ERPs in surgery have been shown the improve outcomes such as patient satisfaction and recovery times Gynaecological cancer patients who undergo CXRT and BT often have substantial recovery needs	Multidisciplinary interventions including: prehabilitation, early social work and nutrition referrals, patient/caregiver education, pre-operative carbohydrate loading, goal directed fluids based on individual patient output, preventative nausea control, and opioid-sparing multimodal analgesia	Multidisciplinary team including a radiation oncologist and anaesthetist	Covering the pre-, intra- and post-operative phases. Number of BT procedures variable depending on delivery of HDR (4-5 times), PDR (1-2 times) and individual patient factors.
Prehabilitation in Locally Advanced Cervical Cancer Patients Receiving Radiotherapy [27]	Prehabilitation intervention was introduced with the goal to enhance patient health and prevent or reduce treatment related impairments	Following baseline functional assessment, patients received physiotherapy, nutritional support and counselling	Not reported	Initiated 2 weeks prior to initiation of XRT until the end of treatment 5 x physiotherapy, 7 x nutritional advice sessions and 7 x psychological counselling
RadBone: bone toxicity following pelvic radiotherapy [28,33] (ongoing study)	Patients treated with XRT are at risk of bone toxicity such as XRT-related insufficiency fractures There is significant cost, morbidity and mortality associated with osteoporotic fragility fractures Previous reports of the benefit of zoledronic acid administration prior to spinal XRT in reducing bone toxicity	Patients undergo a musculoskeletal health assessment and a prehabilitation exercise programme from Prehab4Cancer The tailored exercise regimen comprises RE-HIIT, progressive/continuous aerobic training, and resistance training targeting large muscle groups. Training zones are calculated using age-adjusted heart rate formulae. Nutritional support uses the PG-SGA screening tool, with patients receiving nutritional advice, weight monitoring, and referrals to dietitians, dependant on risk. Well-being support includes assessments like EQ5D, EORTC QLQ-C30, WHODAS, and Self-Efficacy Scale, with referrals to mental health services or liaison with clinical teams if necessary Bone health is evaluated using FRAX and baseline DEXA scans, with treatments aligned with UK recommendations, including educational leaflets from the Royal Osteoporosis Society and supplements Observational arm receives usual care and investigations	Prehab4Cancer’s exercise specialists receive training in nutrition, cancer rehab, and Sage and Thyme foundation communication skills Local instructors are Level 3 qualified or have Level 4 cancer or pulmonary rehab qualifications and have shadowed specialists in clinical settings	Personalised prehabilitation exercise package during XRT, from initial assessment continuing for 12 weeks Bone health: Medium risk or above - calcium (1000 mg once daily) and vitamin D (800 IU/day) High risk - alendronate 70mg once weekly or annual intravenous zoledronic acid infusion

*Abbreviations:* BT =  brachytherapy, CXRT =  chemoradiotherapy, DEXA =  dual-energy x-ray absorptiometry, EORTC QLQ =  European Organisation For Research And Treatment Of Cancer (EORTC) Core Quality of Life Questionnaire, FRAX =  fracture risk assessment, HIIT =  high-intensity interval training, XRT =  radiotherapy, ERP =  enhanced recovery pathway, HRQoL =  health related quality of life, HDR =  high dose rate, PA =  physical activity, PG-SGA =  patient generated subjective global assessment, PDR =  pulsed dose rate, WHODAS =  World Health Organisation Disability Assessment Schedule

**Table 4:** Overview of the studies evaluating unimodal physical exercise interventions using a modified TIDieR checklist.

Study	Why	What	Who	When and how much
Strengthening of Pelvic Floor Muscles for Incontinence in Cervical Cancer [38,42]	UI is an issue commonly experienced by cervical cancer patients potentially caused by dysfunction PFM and neural controls influencing storage and voiding XRT also causes actinic injuries contributing to PFM dysfunction Prehabilitation with PFMEs to preserve or increase PFM strength may prevent or reduce UI	Participants received instruction using a pelvic model to demonstrate pelvic floor muscle contractions. Repeat demonstrations were provided, and a pamphlet was offered based on patient preference. The intervention included 4 PFMEs: 1) Kegel exercises 2) squeeze and release 3) pelvic floor/inner thigh ball squeeze 4) lower trunk rotation/lying hip rotation Daily follow-ups were conducted, and compliance was tracked using a logbook.	Not reported	Pre- during and post XRT Period of 12 weeks beginning when patients came for their planning scan PFMEs performed 4x daily (approx. 18-20 minutes for the 4 exercises)
Early Intervention to Prevent Lower Limb Lymphedema of Gynecological Malignancy (NCT05793749) [39] (ongoing study)	Patients may develop lower limb lymphoedema resulting from surgery and XRT. Lymphoedema is difficult to cure and has a significant impact on limb function, psychological well-being and QoL Long-term lymphoedema treatment can be a financial and psychological burden Evidence that early intervention with lymphatic drainage and exercise may prevent lymphoedema	Prophylactic lymphoedema treatment with manual lymphatic drainage, preventative compression stockings after each drainage, skin care and functional exercises. Control group to receive standard care	Lymphoedema therapist	Post-surgery, during XRT Manual lymphatic drainage 2x weekly after the start of XRT (Total of 10 times). Interval of at least 48 hours but < 2 weeks Functional exercises 2x daily (approx. 15-20 minutes each time)
Pre-rehabilitation of the pelvic floor before radiation therapy for cervical cancer: a pilot study [35,40]	XRT is a risk factor for PFM dysfunction Rehabilitation treatments to reduce XRT impact include vaginal desensitisation, pelvic floor re-education and electrostimulation Limited evidence for the use of these in prehabilitation but there is evidence to support PFMEs to prevent UI	Participants taught PFMEs during physical therapy session and provided with educational materials and pre-recorded audio instruction to continue at home 1)Low contractions = 8 maximal voluntary contractions (6s each with a rest of 10s) 2)One-second contractions = 8 maximal voluntary contractions followed by rest 3)The “knack” = a voluntary precontraction of the PF prior to activities that increase intra-abdominal pressure	Physical therapist specialising in oncology and pelvic floor rehabilitation	Pre-, during and post-XRT 30 minute session approx. 1 month pre-XRT instructed to perform 2x daily
Prophylactic complex physiotherapy in gynecologic cancer survivors [34]	Complex decongestive therapy is an effective and non-invasive technique but prophylactic use is not standard of care The exercises chosen were to enhance muscle function and increase lymphatic drainage by facilitating movement of the lymphatic fluid in the interstitial compartment to the lymphatic system	All patients were informed about lower extremity lymphoedema, preventive complex physiotherapy and skin care The physiotherapy included manual lymphatic drainage massage, a home exercise program (breathing and lower extremity exercises), and compression stockings. Patients who declined prophylactic physiotherapy formed the control group receiving usual care	Gynaecology oncologist and physiotherapists	Referred to the physiotherapy unit at first post-operative visit prior to adjuvant treatment (Pre-XRT) Duration not reported 5x per week
Exercise during treatment for advanced cervical cancer [37]	Patients with gynaecological cancers often present with cancer related fatigue, reduced cardiorespiratory capacity and lower activity levels PA is linked to improved health outcomes in cancer patients but there is a gap in evidence for PA during treatment and in cervical cancer patients Exercises were chosen to provide a full body workout with additional attention given to the lower limbs to reduce functional impairment	During the familiarisation phase the trainer performed initial assessments, taught the exercise techniques and worked on postural control The exercise programme consisting of strength (supervised) and cardiorespiratory (unsupervised) training	Researcher with a background in in sports science, exercise physiology and nutrition	Pre-, during and post CXRT 2 week familiarisation period prior to CXRT, 6 week intervention finishing up to 2 weeks post-XRT 2x weekly strength training (7 exercises) -exercise prescription varied, e.g., 2 sets of 8 reps per exercise an intensity of 3–1 RIR compared to 2 sets of 15 reps at 7–4 RIR post CTx Cardiorespiratory training was 90-180 minutes of walking weekly at 65-85% theoretical maximum HR
OnkoFit II (NCT04517019) [36,41] (ongoing study)	PA is linked to improved HRQoL and has been shown to be feasible during XRT but further trials are required to optimise strategies and timing The goal is to test a PA programme that requires minimal resources and can be implemented easily into current workflow as 1:1 PA programmes have geographical and resource limitations Activity trackers are easy to use and provide real-time data that may improve self-awareness and motivation 6000-step threshold based on literature search indicating that a sedentary lifestyle was defined by this threshold	Baseline step measurements form the basis of personalised weekly step goal Arm A: Exercise advice and educational booklet Arm B: Exercise advice, booklet and activity tracker with no step goal Arm C: Exercise advice, booklet, activity tracker and personalised step goal calculated from baseline measurement and increased from previous week’s average by 10% each week	Not reported	Pre-, during XRT Baseline steps for step goal calculated for approx. 7 days from consent to XRT planning scan Arm A and B: 1.5 hours of moderate PA or 75 minutes of strenuous PA weekly Arm C: as A and B but with additional weekly step goal up to 6000 steps during XRT

*Abbreviations*: CTx =  chemotherapy, CXRT =  chemoradiotherapy, HR =  heart rate, HRQoL =  health related quality of life, PA =  physical activity, PFM =  pelvic floor muscles, PFME =  pelvic floor muscle exercise, QoL =  quality of life, RIR =  reps in reserve, UI =  urinary incontinence, XRT =  radiotherapy

**Table 5:** Overview of the studies evaluating unimodal psychological interventions using a modified TIDieR checklist.

Study	Why	What	Who	When and how much
A nurse led psychosocial intervention with peer support to reduce psychosocial needs in women with gynaecological cancer [46,69]	Timely provision of self-care information for adverse effects is associated with better coping, reduced fear of sexual intercourse and improved compliance with vaginal rehabilitation Peer support can increase patient satisfaction through addressing patients’ needs and providing psychosocial support The nurse led intervention was designed to address patients psychosocial, psychosexual and physical needs using tailored information to improve recall and an orientation element based on evidence on preparing patients for potentially threatening medical procedures	Trained nurses led face-to-face or telephone consultations. Session 1: Patients had XRT facility tour followed by a consultation to address their top 3 concerns prior to XRT and coaching on self-care for side effects and stress reduction offered. Session 2: Discussion about XRT side-effects and normalising fears. Coaching in interventions to address adverse effects and optimising vaginal health. Session 3: Focus on vaginal health and psychosexual recovery including how to approach resuming sexual activity Peers matched by diagnosis, treatment, and age. Peers offered psychosocial support, encouraged adherence to self-care, and referred complex issues back to the treatment team	CNS and peers (gynae cancer survivors ≥ 2 years post-XRT) Both attended relevant training workshops	Pre-, during and post- XRT Approx. 12 weeks 3 nurse consultations (pre-treatment, mid-treatment, end-of-treatment) (approx. 30-60 minutes) 5 peer support consultations (phone) (pre-treatment, mid-treatment, end-of-treatment, 2 and 4 weeks post-treatment)
PeNTAGOn [48,49,68]	Patients with gynae cancer undergoing XRT experience distressing side-effects that impact psychosocial functioning and relationship Comprehensive treatment preparation that addresses patients’ informational, physical and psychological needs may reduce distress Intervention based on pilot study and patient feedback Nurses use an XRT prompt sheet developed using the literature and patient/professional input as a guide to tailor the intervention	Session 1: XRT unit tour and consultation. Misconceptions clarified, distress assessed, and controlled breathing exercises with positive self-talk taught. The nurse discusses supportive care needs, vaginal health, psychosexual rehabilitation, menopause, and infertility. Self-care coaching and tailored information are provided. Session 2: Focus on side effects and self-care strategies, including vaginal dilator use and PFMEs. Patient’s treatment experience, fears, and peer call feedback are assessed. Self-care barriers and stress-reduction strategies discussed. Session 3: Addresses anxiety about XRT completion, ongoing issues, side effects, and resuming sexual activity. Provides survivorship care plan. Session 4: Discusses post-treatment concerns, self-care barriers, vaginal dilator use, and elicits any new concerns Peer provides psychosocial support and normalises patient’s emotions and reactions. Encourage adherence to self-care Usual care - regular assessments by a nurse and/or radiation oncologist throughout treatment	CNS and matched peers (gynae cancer survivors ≥ 2 years post-XRT) Both attended relevant training workshops	Pre-, during and post- XRT 4 nurse consultations -face-to-face/phone (pre-, mid-, end-of- and post-treatment) (approx. 30-60 minutes) 4 peer-led support sessions – phone (pre-, mid-, end-of- and post-treatment) (approx. 30 minutes)
A study to assess effectiveness of social support group among cervical cancer patients and their caregivers [61,78]	Studies have shown cancer support groups can improve QoL and coping The intervention protocol was validated by experts in nursing and radiation oncology then piloted with a small group	Groups formulated for patients (and their caregivers) having XRT at a similar time Sessions discussed cancer, treatment, and management of XRT side effects. In the 3^rd^ session the group were taught stress management and relaxation strategies Comparison group received routine care	Not reported	During XRT 30 minute meetings, 1x weekly for 3 weeks
A Randomized Controlled Trial on Pranayama and Yoga Nidra for Anxiety and Depression in Patients With Cervical Cancer Undergoing Standard of Care [59,77]	Yoga Nidra promotes deep relaxation, while Pranayama is a self-managed breathing practice suitable for all ages and genders to support physical and mental health	3 component yoga intervention: 1) breathing exercises, 2) Pranayama, 3) Yoga Nidra relaxation	Yoga professional	During XRT 30 minute yoga intervention, 2x daily, 5x per week for 6 weeks
A randomized trial of the effect of training in relaxation and guided imagery techniques in improving psychological and quality-of-life indices for gynecologic and breast brachytherapy patients [60]	Psychological interventions such as relaxation and behavioural therapy have been effective in reducing the emotional and psychological burden associated with CTx and other medical procedures Relaxation is a low cost and accessible intervention	All patients informed about BT and discussed concerns Experimental group received additional relaxation and guided imagery training and were provided with a tape to use during BT. The recording instructed patients on full body relaxation and breathing techniques followed by guided imagery based on information provided by the patient prior to recording	Not reported	Pre- and during BT Intervention delivered 1-2 weeks prior to hospitalisation The first part of the intervention was 45-50 minutes. Experimental group received an additional training on relaxation and guided imagery
An effective group psychoeducational intervention for improving compliance with vaginal dilation: A randomized controlled trial [44,52]	XRT and other treatments can cause significant changes to the vagina and other distressing side-effects, e.g., treatment induced menopause Compliance with vaginal dilation and use of lubricants was hypothesised to reduce side-effects of XRT Intervention was based on the information motivation behavioural skills model Time was made available during the sessions for peer discussion on sexuality to help participants overcome reservations relating to new behaviours (e.g., dilator use) and address concerns about resuming sexual activity	Control group received a brief session on vaginal damage from XRT and use of dilators and lubricants, with partners allowed The psychoeducational group participated in small groups sessions (excluding sexual partners) and a follow-up call to problem solve unexpected issues relating to dilator use Session 1: Introductions and goals overview. Covered body image, cancer myths, female anatomy, XRT effects, Kegel exercises, vaginal dilators, and menopause symptom control. Homework: practice Kegel exercises, use dilators (if no contraindication), examine genitals with a mirror, and pre-read Session 2 notes. Session 2: Reviewed homework and discussed dilator use and sexual misconceptions. Addressed sexuality in later years, female sexual response, radiotherapy and menopause effects, resuming sexual activity, and normalising feelings. Concluded with a wrap-up and resources on sexuality	Control – XRT nurse or XRT technician Experimental – Clinical psychologists and XRT oncology nurse	During and post-XRT Control – 30 minute session in last week of XRT Experimental – 2 x 2 hour sessions over 1-2 weeks during XRT and a telephone session 3 weeks post-XRT
Assessment of an onco-sexology support and follow-up program in cervical or vaginal cancer patients undergoing brachytherapy [70]	XRT and BT can induce long-term vaginal changes which can impact patients’ physical and psychosexual well-being Patients value their sexuality but it is rarely addressed during routine care and follow-up	The experimental group received a 3-step sex therapy support programme. Step 1: The nurse discusses BT service and collects personal details including details relating to sex. Step 2: During hospitalisation, side-effects of BT their impact on sexuality are discussed. Patients are taught the concept of vaginal re-education and vaginal dilators. Step 3: Body image and sexual function are evaluated. Then importance of vaginal dilatation is reiterated Control group received usual care including explanation of BT side-effects and dilator use at the initial consultation with their physician and hospitalisation	A dedicated nurse with a degree in sexology	Pre, during and post BT 3 sessions - at initial consultation prior to brachytherapy, during hospital stay for BT and at 2 month follow up
Effect of Foot Reflexology and Aromatherapy on Anxiety and Pain During Brachytherapy for Cervical Cancer [62]	Aromatherapy uses essential oils to create a mind-body connection, releasing endorphins and serotonin. Reflexology stimulates neural pathways to manage pain and enhance body function These safe and cost-effective methods may complement conventional treatments, potentially improving pain and anxiety	In the experimental group, practitioners introduce themselves, explain reflexology and instruct the patient to focus on slow, deep breathing Each session begins with relaxation techniques, including ankle-loosening, range of motion exercises, metatarsal scrubbing, spinal stroking, and foot rocking. This is followed moderate pressure reflexology, focusing on key reflex points. An essential oil diffuser is placed at the patient’s bedside, diffusing the chosen scent until discharge	2 mental health clinical nurse specialist trained in reflexology	During BT period During planning period after BT applicator placement 5x total, 30 minutes per reflexology session
Effect of guided imagery relaxation on anxiety in cervical cancer: randomized clinical trial [45,67]	Relaxation techniques with guided imagery are low-cost, safe and easily applied Utilises visualisation to promote neurophysiological responses and has been shown to improve QoL and physical and psychological well-being when combined with drug treatment Simulation technology can provide immersive and interactive experience	Relaxation and guided imagery using a VR technique was conducted in a private, quiet room. Individuals could choose an interactive video and were instructed on how to use the equipment Step 1: initial relaxation. Step 2: controlled breathing and muscle movements. Step 3: imagery with sensory guidance and alternating slow and deep breathing. Step 4: focusing on their body and returning to normal environment. Control group received usual care	Researcher – qualifications not reported	During XRT 3 x weekly (4 weeks, 12 sessions total) Approx. 10 minutes per video
Effectiveness of an Interventional Package on the Level of Anxiety, Depression, and Fatigue among Patients with Cervical Cancer [65,79]	Guided imagery and muscle relaxation has been used during CTx to reduce anxiety An interventional package was used instead of a singular intervention to better address the complex experience of cervical cancer patients The conceptual framework was Orem’s self-care deficit theory	The interventions utilised JPMR, counselling, self-care and side effect advice Comparison group received routine care	Researcher – qualifications not reported	During XRT JPMR instructed 7x during 4.5 weeks of treatment Counselling 1^st^ and 3^rd^ week Information booklet 1^st^ and last week
Effectiveness of Yoga Nidra in Mitigating Stress in Women Undergoing Curative Radiotherapy for Cervical Cancer [64]	Yoga is effective in reducing physiological and psychological stress Yoga Nidra is easy to learn and requires no asanas so is less physically strenuous for cancer patients and can be practiced safely without supervision	Intervention group received in-person teaching followed by independent practice using pre-recorded verbal instruction Individuals learn to actively alter their states of consciousness and respiratory rate. The practice involves steps such as relaxation, setting a resolve, rotating consciousness through the body, breath awareness, image visualisation, and repeating the resolve	Principal investigator - Yoga practitioner	Pre- and during XRT Initial 23 minute taught session pre-XRT Relaxation exercises 2x daily, 5x per week for 4 weeks (duration of XRT)
Effects of Integrated Music-Video Therapy on Pain and Anxiety During High-Dose-Rate Brachytherapy [71]	Music therapy has been reported to relieve pain and anxiety in individuals with cancer and painful procedures Previous research has suggested that integrated music-video therapy is more effective at distracting participants from pain than music therapy alone Music-video therapy is a non-invasive approach that can be administered easily and safely in a BT environment Nature videos were chosen based on previous research suggesting watching natural scenery reduces anxiety and stress Songs were chosen based on each participant’s preference due to suggestion in the literature that tailored music is more effective than generic	The video was projected onto the ceiling to allow the individuals to watch whilst lying on the therapy bed Music selected according to the participant’s preference of genre was run simultaneously with the video Participants were provided with headphones and could control volume Genres included folk, Christian (classic and contemporary), Buddhist hymns, pop, Korean trot, ballads, Korean traditional and meditation	Researcher monitored participant remotely during therapy Video production specialist involved in video selection and content validity	During BT Individuals were randomised to receive the music therapy during sessions 1-3 or 4-6 of BT Music played for approximately 40 minutes (beginning – end of BT session)
Effects of mindfulness-based stress reduction on cervical cancer patients undergoing concurrent radio-chemotherapy [63]	Patients with cervical cancer often experience significant physical and psychological burden associated with illness and treatment Mindfulness based relaxation has been shown to have a positive impact on negative emotions, self-perceived burden, sleep and CRF	In addition to the usual nursing care, the observational group engaged with mindfulness based relaxation, each week reviewing the previous week’s work and daily practice at home Week 1: Patients introduced, taught mindfulness-based stress reduction including mindful breathing and discussed any doubts. Week 2: Practiced meditation with classical music, reflecting on feelings. Homework: As previous + meditation exercises. Week 3: Practiced body scan techniques with classical music, focusing on sensations Week 4: Practiced walking meditation with classical music, focusing on body movements and ground contact. Week 5: Taught eight-sectioned exercises. They practiced the first 3 sections, noting changes in emotions and sensations. Week 6: Practiced emotional regulation through meditation and mindful breathing	Not reported	During XRT 6 weeks, 2 hour in-person session and daily at-home practice Teaching hours: Monday-Saturday at 2 different times. Patient choice of hours depending on their capacity
Humanity Assurance Protocol in Interventional RadiotheraPY (HAPPY) [50,58,76]	Diagnosis and treatments such as BT can induce significant anxiety and distress Each recommendation/ intervention addresses an identified cluster of needs/issues expressed by patients in a preliminary study	During BT patients could select music, videos, paintings, psychological support and/or prescription anxiolytics as necessary. Information booklets with FAQs were given prior to BT. Reassuring and familiar terms were used frequently. External genital depilation was done at home. A bladder catheter was fitted shortly before the procedure to minimise in-situ duration and discomfort. An operator was present during positioning and plan optimisation using proximity and touch for reassurance and minimise feelings of isolation	Multiprofessional group including oncologist and RT technician	Pre- and during BT
Impact of an Educational Physiotherapy-Yoga Intervention on Perceived Stress in Women Treated With Brachytherapy for Cervical Cancer (KYOCOL) (NCT06263283) [72] (ongoing study)	Individuals with cervical cancer undergoing BT are at risk of experiencing increased anxiety Yoga is a mind-body practice that can reduce stress in individuals with cancer Recent literature review indicates need for more non-pharmacological interventions to support patients receiving BT Previous research has demonstrated feasibility of a physiotherapy-yoga intervention with patient education for individuals with breast cancer	The control group will receive standard care according to their centre (psychological support, physiotherapy and dietetic input can be suggested as necessary) The experimental group receive yoga sessions supervised by a physiotherapist Option for patient to also continue to practice using the educational tools provided	Physiotherapist	During and post-BT Sessions at 3 timepoints during the course of BT Optional additional autonomous practice throughout treatment and up to 15 days post-BT
Impact of early institution of palliative care on quality of life of patients with locally advanced cancer of the uterine cervix - EIPAQ-CX [57,75]	Early integration of palliative care has been used for some patients to address important issues and facilitate shared-decision making to reduce the physical and psychological burden experienced by patients	Patients attended palliative care clinic at pre-, during, post-XRT in addition to additional visits as required by the patient The team managed physical symptoms and provided individual counselling sessions to the patient and their caregiver at their clinic or home where required Caregivers were also educated on prognosis and optimal home-based care strategies	Palliative care team including palliative care doctor, nurse and a social worker	Pre -, during and post XRT/BT Referred pre-XRT, clinic sessions and every 2 weeks during XRT, additional care during weekly XRT review, a session post-XRT/pre-BT, a session 6 weeks post-BT and 3 months post-BT
Music Relaxation Video and Pain Control: A Randomized Controlled Trial For Women Receiving Intracavitary Brachytherapy For Gynaecological Cancer [56,66,74]	Auditory stimulation can engage neurological pathways to lower pain perception Music should have a slow, stable rhythm, low-frequency tones, and soothing melodies for anxiety reduction Effective therapeutic music should be played for 20-30 minutes, twice daily Combining music with visual arts enhances pain reduction	Experimental group participants selected a music relaxation video to watch during BT. The video options provided were selected with attention given to harmonic consonance and tempo, the visual element consisted of peaceful images For each session the video was started shortly before the BT pulse began and was played without interruption	Music therapist selected initial videos for the patient to choose from Videos played in the patient’s room observed by the investigator	During BT Each video was 30 minutes and shown to the patient at 4 intervals (total 120 minutes) Intervention delivered on 2^nd^ day as inpatient at 9am, 1pm and 5pm then on day 3 at 9am
Preservation of Immune Function in Cervical Cancer Patients during Chemoradiation using a Novel Integrative Approach [54,55]	Biofield therapies, e.g., HT, may affect disease processes by inducing relaxation, which reduces stress responses and enhances immune function HT aims to restore energy balance, facilitating self-healing through a caring relationship between practitioner and patient Limited research suggests reductions in pain, distress, fatigue, and improved QoL particularly in cancer patients	The experimental group received HT sessions including: 1) grounding and centring to support healing and immune function, 2) pain drain to reduce energy congestion and detoxify the liver, 3) chakra connection to balance energy centres, 4) magnetic unruffling to clear congested energy and distress, and 5) mind clearing for relaxation and focus. Some techniques involved physical touch. The relaxation therapy control group received sessions guided by staff using scripts for progressive relaxation, autogenic relaxation, and imagery-based relaxation The additional control group received usual care	3 nurses certified healing touch practitioners Relaxation therapy was delivered by 3 primary therapists (trained research assistant or graduate students)	During CXRT HT (20-30 minutes) or relaxation (20-25 minutes) interventions were done 4x per week after each XRT session on non-chemotherapy days
Regular counselling by an oncology nurse increases coping with side effects during outpatients radiotherapy of gynecological malignancies [53]	Not reported	In the experimental group patients and their relatives received a nurse-led counselling session including discussion of XRT-related side effect, sexuality and psychosocial concerns	Oncology nurse	During XRT 3x 60 minutes sessions
SISTER [73] (ongoing study)	In the USA, Black women are at an increased risk of being diagnosed with high-risk endometrial cancer and experience poorer overall 5-year survival rates Social isolation contributes to poor overall survival in individuals with cancer and is an identified sub-issue within distress Black women with cancer are at an increased risk of social isolation due to systemic and structural stressors Previous research suggests interventions such as culturally relevant peer support groups can be effective in reducing social isolation in Black women with breast cancer The intervention arms were developed using a multi-level process including a systematic review of relevant literature and input from multiple stakeholders	The enhanced usual care group receive information on local support groups recommended by The National Comprehensive Cancer Network. Written materials are from the ECANA relating to coping with side effects and establishing support structures and healthy behaviours during treatment The facilitated virtual support group will include group conversation and structured topics (e.g., side effects, nutrition, mental wellbeing, finances and family dynamics) with facilitated discussion relating. The topics will be consistent between each group The peer support group receive 1:1 sessions via telephone timed to be close to or during a treatment visit. Session content is tailored to the needs of the individuals and focuses on social support	Facilitated support group: a trained ECANA peer supporter and a co-facilitator (a professional trained in either nutrition, psychotherapy, cognitive behavioural therapy, or medicine) Peer support: ECANA peer supporter	Pre and during treatment (XRT, CTx or immunotherapy) Facilitated support group: weekly meeting 1:1 peer support: calls are no more frequent than 1x weekly and no less frequent than 1x every 3 weeks (allows for treatment schedule variation)
Using Reiki Therapy to Improve Symptoms Associated With Brachytherapy in Patients With Gynecological Malignancies (Reiki-Brachy) [43] (ongoing study)	Reiki is an energy healing practice with the goal to promote relaxation and physical, emotional and spiritual well-being	In a quiet clinic room with calming music and optional aromatherapy, the Reiki therapist places their hands on or near the patient’s body, using various hand positions to align with energy centres. The therapist channels energy into the patient, who may feel warmth, tingling, or relaxation. Techniques include different hand movements and visualisation	Reiki therapist	During BT 1 session between placement of BT device and first treatment
Yoga Therapy During Chemotherapy and Radiation Treatment for the Improvement of Physical and Emotional Well-Being in Patients With Stage IB2-IIIB Cervical Cancer (NCT04622670) [51] (ongoing study)	Yoga practice promotes mind-body connection through meditation and controlled breathing	Patients in the intervention group attend yoga classes throughout CXRT followed by a yoga manual and video post-treatment The waitlist control group receive usual care and are offered group yoga classes 3 months post-CXRT	Yoga therapist with 10 + years’ experience	During CXRT 2 x 60 minute classes per week, 5-6 weeks, up to 15 classes during CXRT

*Abbreviations*: BT =  brachytherapy, CNS =  cancer nurse specialist, CRF =  cancer-related fatigue, CTx =  chemotherapy, CXRT =  chemoradiotherapy, ECANA =  Endometrial Cancer American Network for African-Americans, FAQ =  frequently asked questions, HT =  healing touch, JPMR =  Jacobson Progressive Muscle Relaxation, PFME =  pelvic floor muscle exercise, VR =  virtual reality, XRT =  radiotherapy

**Table 6:** Overview of the studies evaluating unimodal nutritional interventions using a modified TIDieR checklist.

Study	Why	What	Who	When and how much
A phase 2 randomized controlled trial of oral resistant starch supplements in the prevention of acute radiation proctitis in patients treated for cervical cancer [98,99]	Acute radiation proctitis, occurring during or soon after XRT, is due to epithelial and vascular damage, while chronic radiation proctitis manifests months or years later, characterised by ischemia and bleeding Reducing rectal dose may reduce toxicity but can be difficult with conventional XRT in low-income countries Increasing amylase-resistant starch intake can boost colonic butyrate, beneficial for gut health. High-amylose maize starch is hypothesised to prevent radiation proctitis by increasing colonic butyrate production, thus reducing inflammation and aiding mucosal healing	The experimental group were given high-amylose maize starch Control group received a placebo containing commercially available maize starch Both were mixed with water or milk	Study dietician supervised administration of the starch	During XRT 30g of experimental or placebo starch mixed with 150ml of liquid 2x daily for 6 weeks (duration of XRT + BT)
A Randomized, Double-Blind Pilot Trial of Hydrolyzed Rice Bran versus Placebo for Radioprotective Effect on Acute Gastroenteritis Secondary to Chemoradiotherapy in Patients with Cervical Cancer [90]	Animal studies indicate HRB significantly reduces inflammation in colitis models. It may also inhibit mast cell degranulation and cytokine production A human study has suggested potential anti-inflammatory benefits HRB may reduce the effect of XRT-related intestinal inflammation	The experimental group received HRB Control group received a dextrin based placebo	Not reported	Pre- and during XRT 3 packets of HRB (1g of HRB per packet) or placebo 3x daily, starting up to 1 week pre-CXRT until the end of XRT
Arginine, glutamine, and fish oil supplementation in cancer patients treated with concurrent chemoradiotherapy: A randomized control study [83]	CXRT related toxicities may result in poor outcomes due to unplanned breaks, increased overall treatment time and incomplete treatment Supplements including arginine, glutamine, and omega-3 fatty acids may improve immune health and have been linked to reduced post-operative complications in some cancer patients	The standard diet group received nutritional counselling throughout XRT with a calorie and protein recommendation The experimental group received arginine, glutamine, and fish oil supplementation through formula and sachets	Dietician, nurse and a doctor	During XRT Nutritional formula of fat 28.5 g/L from corn oil, medium chain triglyceride, and fish oil and protein 61.5 g/L from casein, arginine, and glutamine 2 glasses per day (1 glass = 250 mL: 250 kcal), 1 hour pre- and post-XRT session and supplement sachets over the weekend
Changes of immune response and side effects before and after nutritional intervention in cervical cancer patients with concurrent chemoradiotherapy [114]	Nutritional status can impact CXRT toxicities	The comparison group received standard nutritional support, e.g., avoiding foods with a high fat content The intervention group were assessed and treated according to nutritional status – patients with a good nutritional status were advised to continue regular diet, whereas patients with suspected or confirmed malnutrition were given tailored nutritional advice including calorie and macro goals	Not reported	During and post-XRT Started at the same time as CXRT until 1 week post-treatment Malnourished group recommended 84-126 kJ/(kg-d), 15-20% protein, 25-30% fat and 50-60% carbohydrates
Decreasing the Adverse Effects in Pelvic Radiation Therapy: A Randomized Controlled Trial Evaluating the Use of Probiotics [81]	Germ-free mice resist radiation enteritis, suggesting gut microbes affect radiosensitivity Increasing lactobacilli intake in experimental animals reduces bacterial translocation and inflammation Probiotics may enhance mucus production, improve barrier function, and modulate microbiota, potentially preconditioning the gut to be resistant radiation-induced injury	HDP and LDP groups given capsules containing *Lactiplantibacillus plantarum* HEAL9 and *Lactiplantibacillus plantarum* 299 with maltodextrin filler Control group given placebo capsules An appointment was scheduled halfway through XRT to remind patients to take the product and fill out the daily GI health diary	Not reported	Pre-, during and post- XRT 2 capsules daily from 1-2 weeks pre-XRT until 2 weeks after XRT (XRT 23-26 days) 1:1 bacterial strain -total dose of 1 × 10^10^ CFU/capsule (LDP) or 5 × 10^10^ CFU/capsule (HDP)
FIDURA [80,102] (ongoing study)	Based on the hypothesis that increased dietary fibre during XRT may reduce long-term GI issues by maintaining intestinal mucus layers and preventing gut-wall starvation, which otherwise may lead to inflammation and chronic conditions Mobile applications are easily accessible for patients and facilitate direct transfer of individual nutritional data for analysis Capsules given instead of powder for palatability	All participants given dietary advice including recipes and fibre goal Experimental group receive additional fibre via psyllium husk capsules Control group receive placebo capsules containing maltodextrin with no additional fibre Use mobile application to track dietary fibre	Dietician	Pre-, during and post-XRT 16g of dietary fibre from food 5.5g of additional dietary fibre in psyllium capsules 15 capsules (5 x 3) daily from 2 weeks pre-XRT until approx. 4 weeks post -XRT
Dietary regime during radiation therapy for carcinoma of the uterus [104]	Diet designed to reduce faecal residue during BT to avoid disturbing treatment due to bowel motions The bland, low-fibre diet to reduce irritation of the bowel due to XRT-related inflammation	Patients received LRD during BT followed by an increased but bland, low-fibre diet	Not reported	Pre-, during XRT/BT and post-XRT LRD 2 days before BT and until radon source removed (approx. 6-12 days) Bland, low-fibre diet for 4 weeks until end of XRT and resolve of acute side effects Daily intake of 2000 calories with 90g of protein
Effect of an Anti-inflammatory Diet on Patients with Cervical Cancer [94,112]	GI toxicities can leave patients at risk of malnutrition and micronutrient deficiencies Probiotics have shown anti-inflammatory effects in inflammatory bowel disease, while micronutrients and trace elements offer antioxidant and immune-modulating benefits during intestinal inflammation. These may reduce inflammation and symptoms in PRD LRD is used to reduce XRT-induced diarrhoea but does not prevent malnutrition	Control group following LRD Experimental group following AID including foods containing immune modulating ingredients, e.g., Omega-3 fatty acids, antioxidants, soluble fibre and probiotics In-person and written dietary advice individualised according to comorbidities and given with consideration of food accessibility in their home area	Nutritionists	Pre-, during and post CXRT and BT 5 appointments: 2 weeks prior to treatment, at the start of CXRT, during the 3rd cycle of CTx, at the end of BT and 3 months after completion AID = 28-31 kcal/kg/day, based on 30-40% fat, 20% protein, and 40-50% carbohydrates LRD = 28-31 kcal/kg/day, based on 20% fat, 20% protein, and 60% carbohydrates, ≤ 20g fibre and ≤ 5g of lactose
Effect of Probiotics for the Prevention of Acute Radiation-Induced Diarrhoea Among Cervical Cancer Patients: a Randomized Double-Blind Placebo-Controlled Study [93]	Research suggests that probiotic supplementation may prevent or reduce RID Potential mechanisms, include correcting dysbiosis, reducing intestinal inflammation and apoptosis, upregulating the gut immune response, and aiding lactose digestion *Lactobacillus* and *Bifidobacterium* are commonly used strains in previous studies	All participants received a pamphlet with standard dietary recommendations The intervention group received capsules containing *Lactobacillus acidophilus* LA-5 plus *Bifidobacterium animalis* subsp. *lactis* BB-12 Control group given placebo capsule containing starch	Not reported	During XRT 1 capsule 3x daily for the duration of XRT (5 weeks)
Effectiveness of a nutritional intervention in the reduction of gastrointestinal toxicity during teletherapy in women with gynaecological tumours [100,101]	GI toxicities can impact nutritional status, QoL and may cause patients to suspend or discontinue XRT resulting in poor outcomes FODMAP diet modifies factors that influence radiation-induced enteropathy such as regulation of intestinal motility, reduced lactose intake and osmotic agents	Control group followed standard Mexican diet Experimental group followed FODMAP diet using a nutritional guide	Not reported	During XRT Duration of XRT (approx. 5-6 weeks)
Efficacy of ω-3 supplementation on nutritional status, skeletal muscle, and chemoradiotherapy toxicity in cervical cancer patients [92,103,113]	Weight and skeletal muscle loss are associated with unfavourable outcomes, e.g., high risk of toxicity ω-3 has been associated with modulating the inflammatory response, promoting appetite, and preservation of body weight and skeletal muscle mass	All participants received an isocaloric nutritional supplement due to being at nutritional risk Control group received capsules containing olive oil Experimental group received capsules containing ω-3 (EPA and DHA)	Lead investigator - dietician	During CXRT 4 capsules (total 2.5g of omega-3 – 2g EPA ^+^ 450mg DHA OR 2.5g olive oil) daily for duration of CXRT (average 45 days)
Effect of inulin and fructo-oligosaccharide on the prevention of acute radiation enteritis in patients with gynecological cancer and impact on quality-of-life: a randomized, double-blind, placebo-controlled trial [86,87]	*Bifidobacterium* and *Lactobacillus* bacterial strains offer benefits such as pathogen growth inhibition Rectal biopsies from pelvic RT patients show intestinal damage, potentially exacerbated by microbiota changes Carbohydrates, e.g., inulin and FOS, may stimulate the proliferation of healthy bacteria	Control group received a maltodextrin placebo Experimental group received a fibre mixture (inulin and FOS)	Not reported	Pre-, during and post-XRT Prebiotics started 1 week pre-XRT until 3 weeks post-XRT 6g 2x daily of fibre mixture (1:1 inulin:FOS) or placebo in 200ml of water
Effects of Probiotic Lactobacillus Casei DN-114 001 in Prevention of Radiation-Induced Diarrhoea: Results From Multicenter, Randomized, Placebo-Controlled Nutritional Trial [88]	XRT damages basal epithelial cells, impairing the epithelium’s renewal capacity Oral probiotics have shown effectiveness in conditions like gastroenteritis Probiotic efficacy varies by strain, with *Lactobacillus casei* providing significant functional benefits The probiotic strain DN-114 001 has improved inflammation markers in patients with chronic inflammatory bowel diseases	Participants received a fermented liquid yoghurt containing *L. casei* DN-114 001, ^+^ standard starters *Streptococcus thermophilus* and *Lactobacillus delbrueckii* subsp. Bulgaricus (experimental), or a matching sterilised placebo Dietary guidelines, including lists of allowed and restricted foods were given. Fermented yoghurts and other dairy products were prohibited, but other dairy-derived foods were allowed	Not reported	Pre- and during XRT 1 week pre-XRT and throughout treatment course Intervention group received 96ml 3x daily containing 10^8^ CFU/g of *L. casei* DN-114 001, in addition to the standard starters OR same amount of sterilised product
Efficacy of glutamine in the prevention of acute radiation enteritis: a randomized controlled trial [105]	Glutamine supports intestinal health, serves as a precursor to the antioxidant glutathione, modulates immune responses, and aids in cell protection and apoptosis regulation Studies on its efficacy in preventing ARE have shown mixed results The selected dose of glutamine follows previous studies involving XRT and chemotherapy	Participants received a powder to be dissolved in water of either oral glutamine (experimental) or whole casein (placebo) Participants with kidney disease were advised to adjust their protein intake with consideration of the added contribution from the study product	Dietician	Pre- and during XRT 3x 10g sachets daily dissolved in 200ml of water beginning 3 days pre-XRT until completion
Effect of symbiotic supplementation on fecal calprotectin levels and lactic acid bacteria, Bifidobacteria, Escherichia coli and Salmonella DNA in patients with cervical cancer [85]	Synbiotics, containing prebiotics and probiotics, promote health benefits including immunomodulation and intestinal integrity maintenance. Prebiotics like inulin and oligofructose stimulate beneficial bacteria growth, inhibiting pathogenic bacteria and reducing inflammation Synbiotics reduce inflammation in conditions like ulcerative colitis and ARE, with prebiotics such as oligofructose-enriched inulin showing early reduction in FCP (inflammation marker)	Patients received a gel containing either a synbiotic mixture of *Lactobacillus acidophilus* NCFM, *Bifidobacterium lactis* Bi-07 and blue agave inulin (experimental) or a placebo Diet was monitored every 15 days to ensure no diet modifications were done throughout the intervention	Supervised by nutritionists	During XRT Synbiotic contained: 1 x 10^7^ CFU/g biogel of *Lactobacillus acidophilus* NCFM, *Bifidobacterium lactis* Bi-07 1 x 10^6^ CFU/g biogel, and blue agave inulin 3x 20g gel daily for 7 weeks
Multicenter, Phase 3 Trial Comparing Selenium Supplementation With Observation in Gynecologic Radiation Oncology [96]	Early evidence suggests Se may alleviate chemotherapy and XRT toxicity, possibly by increasing antioxidant capacity through enhanced biosynthesis of enzymes that may neutralise XRT-induced hydroperoxides and free radicals in the intestinal mucosa Se may stimulate mucosal repopulation mechanisms but XRT can exacerbate Se deficiency Dose and scheduling chosen based on efficacy and tolerability in previous trials	Experimental group received oral Se supplementation Control group received no supplementation	Not reported	During XRT 500μg of Se (inorganic sodium selenite) orally on XRT days and 300μg of Se on non-XRT days until the last day of XRT
Phase II Study Assessing the Feasibility of Using Elemental Supplements to Reduce Acute Enteritis in Patients Receiving Radical Pelvic Radiotherapy [84]	Studies have suggested that elemental diets can prevent ARE in pelvic XRT patients Elemental diets may produce less free radicals, and are associated with reduced superoxide dismutase or xanthine oxidase activity in the intestine which could minimise XRT damage by preventing free-radical build up 2-3 servings chosen to minimise non-compliance due to disease/XRT-related nausea or bloating	A cohort of patients receiving radical pelvic XRT (without ES) were used for baseline ARE data All participants given XRT diet sheet (low fibre, low lactose, moderate fat) and ES group given instruction on ES diet ES = low osmolar hydrolysate that comprises partially hydrolysed whey/meat/soy protein, safflower oil, medium chain triglycerides, and cornstarch carbohydrate forms	Dietician and radiation oncologist	Pre- and during XRT Low osmolar hydrolysate (460 mOsm/kg water). Calories per 250 ml varies from 1,050 J - 1,260 J 2-3 servings (1 serving = 39g of vital powder 250ml water) daily from 3 days pre-XRT until the end XRT (5-6 weeks)
Preservation of intestinal integrity during radiotherapy using live *Lactobacillus acidophilus* cultures [97]	Intestinal mucosa relies on gut bacterial flora and luminal contents for nutrients but XRT alters flora, mucosal cell permeability, and intestinal motility Lactic acid bacteria, e.g., *Lactobacillus*, has been suggested to prevent diarrhoea caused by pathogenic bacteria and its’ bacteriocin production may help balance microflora during XRT *Acidophilus* strain used was tested for radiation stability	All participants received dietary counselling emphasising sufficient energy and protein intake, whilst avoiding high-fibre, high-lactose and high-fat diets Experimental group received additional yoghurt product containing live *Lactobacillus acidophilus* (NCDO 1748) The product was treated with lactase to hydrolyse lactose and additional lactulose was added to promote growth of *Lactobacillus acidophilus* (NCDO 1748) in the large intestine	Physician and nutritionist	Pre-, during and post-XRT 150ml of product daily (at least 2 × 10^9^ live *Lactobacillus acidophilus* bacteria) from 5 days pre-XRT to 10 days post XRT
Randomized controlled trial of live lactobacillus acidophilus plus bifidobacterium bifidum in prophylaxis of diarrhea during radiotherapy in cervical cancer patients [82]	Severity of acute bowel reactions may predetermine the degree of chronic bowel changes *Lactobacilli* may help balance the microflora during XRT, potentially preventing or reducing GI toxicity	Participants received either capsules of *lactobacillus acidophilus* plus *bifidobacterium bifidum* (Infloran^®^) (Experimental) or placebo capsules containing magnesium stearate, talc, and purified water Participants received standard XRT dietary recommendations and were banned from eating yoghurt or other dairy foods produced by fermentation	Not reported	Pre- and during XRT 2 × 10^9^ units of *lactobacillus acidophilus* plus *bifidobacterium bifidum* (equivalent to 2 capsules) twice daily before meals, from 7 days pre-XRT until the end of XRT
Repurposing Individualized Nutritional Intervention as a Therapeutic Component to Prevent the Adverse Effects of Radiotherapy in Patients With Cervical Cancer [95]	Malnutrition due to low BMI and weight loss > 5% of total body weight are predictive indicators for developing XRT toxicity Previous studies have demonstrated the benefit of an individualised nutritional intervention in reducing treatment related toxicities in patients receiving XRT for other pelvic malignancies Energy and nutrient intake recommendations based on ESPEN guidelines for cancer patients	Retrospective cohort received standard nutritional counselling including allowed and restricted foods + general recommendations for GI toxicities Experimental group received individualised dietary advice and counselling adjusted throughout XRT according to GI adverse effects the patient experienced and their food tolerance	Not reported	During XRT Counselling weekly (4-5 weeks) Dietary calculation based on established guidelines and formula, suggesting 25 to 30 kcal/kg/day with 1.2 to 1.5g protein/kg/day (20-30% protein, 30-40% fats, 40-50% carbohydrates)
The effect of selenium supplementation on the efficacy of concurrent radiotherapy for cervical cancer: a randomized, double-blind, placebo-controlled phase II clinical trial [89,106]	Se is an essential trace element with significant biological functions in human health Small studies indicate that Se can enhance the effects of XRT whilst potentially reducing their toxicity but further RCTs are required for understanding its effect in cervical cancer patients	Selenium yeast tablets (experimental) or placebo	Not reported	During XRT Selenium yeast tablets (100 μg Se) or placebo twice a day (approx. 5 weeks)
The effect of a low fat, low lactose diet during pelvic radiotherapy [109–111]	Several factors can induce diarrhoea during XRT, including malabsorption of lactose and bile salts Removing lactose from the diet and restricting milk intake may help, as can a low-fat diet to reduce faecal bile salt content and diarrhoea	Control group received standard hospital diet Intervention group prescribed a tailored low fat and lactose diet. Carbohydrates and proteins were increased to compensate for reduced fat Advised to take nutritional supplements containing medium-chain triglycerides if they were unable to maintain their body weight	Dietician	During and post-XRT Diet to be continued throughout XRT until 6 weeks post Control - 80 g (44% of energy from fat) Experimental - maximum of 40g fat and 10g lactose per day
The Effects of Immunonutrition Therapy on the Nutritional Status, Immune Function, and Quality of Life of Locally Advanced Cervical Cancer Patients With Malnutrition: an Open-label Randomized Controlled Study (NCT06349148) [108] (ongoing study)	Malnutrition is a common complication in patients with locally advanced cervical cancer undergoing CXRT Individual with cancer commonly experience issues such as immune imbalances, metabolic abnormalities and inflammation Immunonutrition therapy aims to prevent or correct malnutrition, regulate immune function and reduce inflammatory response through the delivery of specific immunonutrients	Individuals are screened using the Nutritional Risk Screening 2002 tool. Anyone identified as being at nutritional risk are then assessed by doctors and nutritionists using the PG-SGA and Global Leadership Initiative on Malnutrition criteria Experimental group receive enteral immunonutrition (Impact, Nestlé) The active comparator group receive isoenergetic standard oral enteral nutrition (emulsion or Nutrison) All participants receive nutritional education from specialist nurses and nutritionists	Specialist nurses Nutritionists	During CXRT Education upon admission and nutritional support throughout CXRT for a total of 5 weeks Experimental group receive 2 bottles of immunonutrients per day (approx. 700 kcal)
Time-Restricted Eating Versus Nutritional Counseling for the Reduction of Radiation or Chemoradiation Tx Side Effects in Patients With Prostate, Cervical, or Rectal Cancers [91,107] (ongoing study)	XRT results in the generation of reactive oxygen species ultimately leading to lethal double-strand DNA breaks Mitochondrial respiration is a major intracellular source of reactive oxygen species in normal tissues Time restricted eating may reduce the amount reactive oxygen species generated during this process and mitigate DNA damage in normal tissues	Active comparator arm receive nutritional counselling only The experimental arm undergo time-restricted eating All participants meet with a dietician to discuss preventative dietary advice without macronutrient or calorie restrictions Offered weekly meetings with the dietician during XRT and on-demand dietary education through a mobile application	Dietician with 20 + years of experience in radiation oncology	During XRT Time-restricted eating Monday-Friday during XRT for a minimum of 14 hours (6-8 hours before until 4-6 hours after XRT)

*Abbreviations*: AID =  anti-inflammatory diet, ARE =  Acute radiation enteritis, BMI =  body mass index, CFU =  colony-forming capsule, CXRT =  chemoradiotherapy, DHA =  Docosahexaenoic Acid, EPA =  eicosapentaenoic acid, ES =  elemental supplements, ESPEN =  European Society for Clinical Nutritional and Metabolism, FCP =  faecal calprotectin, FODMAP =  low fermentable oligosaccharides disaccharides, monosaccharides and polyols, FOS =  fructo-oligosaccharides, GI =  gastrointestinal, HDP =  high-dose probiotic, HRB =  hydrolysed rice bran, LDP =  low-dose probiotic, LRD =  low-residue diet, PRD =  pelvic radiation disease, RID =  radiation induced diarrhoea, Se =  selenium, XRT =  radiotherapy, ω-3 =  omega-3.

### What interventions are being reported in the literature?

#### Multimodal.

Studies evaluating multimodal prehabilitation utilised multidisciplinary teams to address the complex needs of their target population including: dieticians; physiotherapists; radiation oncologists; and prehabilitation exercise specialists (Table 3). Some of the studies also utilised early referrals or input from mental health services or social work teams within their prehabilitation interventions [25,28,32]. Kaliamurthi *et al.* [27] provided cervical cancer patients with a programme consisting of counselling, nutritional support and physiotherapy. Only one study did not specifically mention including a physical exercise element, this was targeted to patients undergoing brachytherapy so the intervention focused on carbohydrate loading, pain control and psychosocial support [32]. RadBone [28] is an ongoing clinical trial that utilises the resources of an established prehabilitation service to provide tailored exercise, nutritional and psychological support in combination with a targeted musculoskeletal health package.

#### Unimodal – Physical/Exercise.

Of the six studies reporting on a unimodal physical exercise intervention, only two implemented a physical activity programme that resemble those more commonly used in prehabilitation prior to surgery. Of these, one was a case study providing a completely tailored exercise intervention for an individual during treatment [37] and the other is an ongoing clinical trial involving a variety of patient groups undergoing radiotherapy using an activity tracker-based programme [36]. Two single-arm (pre-post) interventional studies focused on teaching pelvic floor muscle exercises (PFMEs) and encouraging participants to perform these exercises at home facilitated by educational materials and/or pre-recorded instructions [35,38]. The final category of physical interventions was targeted towards post-surgical patients prior to adjuvant treatment. These studies focus on prophylactic complex physiotherapy including manual lymphatic drainage, skin care, use of compression stockings, and functional exercises that could be performed at home [34,39].

#### Unimodal – Psychological.

The majority of studies addressing psychological wellbeing (n = 17) [43,51,54,56,58–65,67–69,71,72] incorporated relaxation and stress reduction techniques such as controlled breathing exercises, aromatherapy, progressive muscle relaxation, guided imagery, videos, music, meditation, mindfulness, and stress management coaching. Three of which also evaluated the effect of the complementary therapies: healing touch [54], reiki [43], and reflexology with aromatherapy [62]. In the PeNTAGOn trial, and an earlier pilot study [69], Schofield *et al.* [68] used both specialist nurses and matched peers to deliver a tailored psychoeducational intervention that involved elements of relaxation combined with timely delivery of information and support, to address patients’ concerns and psychosexual needs. Kpoghomou *et al.* [70] and Varre *et al.* [53] also provided nurse-led counselling to support and manage patients’ psychological and psychosexual wellbeing, whereas, Jeffries *et al*. [52] favoured a small group approach with taught elements and peer discussion facilitated by healthcare professionals. In their ongoing trial, Oluloro *et al*. [73] are evaluating the implementation of virtual support groups hosted by a trained peer-support and specialist co-facilitators to promote group conversation and discussion of structured topics including mental well-being, finances and family dynamics. The support group intervention is also being compared against enhanced care including more targeted written information and a 1:1 support intervention with a peer trained by the Endometrial Cancer American Network for African-Americans [73].

#### Unimodal – nutritional.

Twelve (50%) of the studies evaluating nutritional interventions were randomised, placebo-controlled trials. Several of these studies focused on the introduction of probiotics or synbiotics (prebiotics and probiotics) into the participants’ diets. The strain and combination of bacteria used, varied between studies including: *Lactobacillus casei* DN-114 001 with standard starters *Streptococcus thermophilus* and *Lactobacillus delbrueckii* subsp. *Bulgaricus* [88]; *Lactobacillus acidophilus* LA-5 and *Bifidobacterium animalis* subsp. *lactis* BB-12 [93]; *Lactiplantibacillus plantarum* HEAL9 and 299 [81]; *Lactobacillus acidophilus* with *Bifidobacterium bifidum* [82]; *Lactobacillus acidophilus* (NCDO 1748) [97]; and *Lactobacillus acidophilus* NCFM, *Bifidobacterium lactis* Bi-07 with blue agave inulin [85]. Garcia-Peris *et al*. [86] instead opted for a mixture of prebiotics, inulin and fructo-oligosaccharide, to stimulate proliferation of *Lactobacillus* spp and *Bifidobacterium* spp populations.

Two studies provided patients with Omega-3 supplements, with Chitapanarux *et al*. [83] additionally including arginine and glutamine into their supplement regimen. Whereas, Muecke *et al*. [96] and Yang *et al.* [106] provided selenium supplements to cervical cancer patients. Some focused on modifying factors through dietary changes, such as no or low-residue (little to no fibre) [104]; anti-inflammatory [112]; low fat and lactose [110]; or low fermentable oligosaccharides, disaccharides, monosaccharides and polyols (FODMAP) [100] diets. Other studies included glutamine [105], hydrolysed rice bran [90], oral resistant starch [99], immunonutrient [108] or elemental [84] supplements. One ongoing trial is evaluating the effect of time-restricted eating compared to nutritional counselling during radiotherapy [91,107].

#### What are the rationales underpinning these interventions?.

Any identified theories and/or rationales for each study are outlined in Tables 3-6. More broadly, multimodal interventions were introduced to improve outcomes and prevent or reduce treatment-related morbidity. Edbrooke *et al*. [25] identified that gynaecological cancer patients require increased emotional and social support due to high-levels of distress. They highlighted that their target patient population did not meet nutritional and exercise recommendations and had expressed the need for external motivation to drive behavioural changes [25]. Therefore, they used a multidisciplinary team to provide psychological, nutritional and exercise support underpinned by HealthChange^®^ methodologies with features such as pedometers and text prompts to provide additional motivation [25].

Lymphoedema is a chronic process, experienced by many gynaecological cancer survivors, that is associated with physical, psychosocial and financial burden [34,39,115]. Daggez *et al*. [34] and Zou *et al*. [39] both utilise a technique that is currently used for the conservative management of lower extremity lymphoedema but introduced it in a prophylactic setting. The exercise program used by Daggez *et al*. [34] was chosen specifically to induce muscle function and promote lymphatic drainage. Adjuvant radiotherapy is a risk factor for lymphoedema therefore the studies included, or intend to include, post-surgical patients scheduled for radiotherapy. Similarly, as radiotherapy is also considered a risk factor for pelvic floor dysfunction, Jagdish *et al.* [38] and Sacomori *et al.* [35] adopted pelvic floor strengthening techniques more commonly used in a rehabilitation setting but initiated them prior to radiotherapy as a preventative measure. Hauth *et al.* [36] acknowledged that whilst a personal supervised physical activity programme is desirable for prehabilitation, it is not always feasible due to cost, geographical limitations and resources. Instead, they designed a physical activity programme that can be carried out at home, is less resource intensive, and more easily implemented into a busy radiotherapy workflow [36]. The ongoing Onkofit II trial [36] uses a wearable activity tracker to monitor patient activity and provide a motivational element to the intervention. Interestingly, when developing a physical activity programme, Tórtola-Navarro *et al.* [37] altered their exercise prescription relative to the timing of treatment, reducing the intensity of physical activity in the days following chemotherapy to account for the anticipated increase in side-effects experienced by the patient.

Psychological interventions aimed to reduce stress and help patients cope with the complex psychological burden associated with treatment and disease. Relaxation techniques were favoured by researchers due to their safety, accessibility, low-cost and effectiveness amongst similar populations in the literature [60,63,65,67,71]. De Oliveira Santana *et al.* [67] used virtual reality technology to provide a more immersive guided-imagery experience that can be applied in a clinical environment to overcome physical barriers of accessing nature. Of the studies focused specifically on patients receiving brachytherapy, interventions promoting relaxation and pain reduction were particularly prevalent due to the high levels of discomfort and distress experienced by patients. Chi *et al.* [56] introduced combined audiovisual stimulation to modify pain perception and maximise the anxiolytic effects of both music and guided imagery. The music was chosen with consideration of the literature suggesting a slow, constant rhythm with predictable dynamics and harmonic consonance at a tempo, similar to the resting human heart rate, was optimal for relaxation [56]. In a similar study, Lim [71] prioritised music based on individual preference and projected nature videos onto the ceiling to ensure the participant could engage with the intervention whilst lying on the therapy bed. Several studies particularly emphasised the relevance of mind-body connection as a core element of the intervention to promote relaxation [51,59,62,64,67,72]. Tagliaferri *et al.* [58] took a multidisciplinary approach to assess the needs of brachytherapy patients from different perspectives and developed a number of interventions and recommendations to address the needs and concerns expressed by patients. As an example, they allowed patients to select music and relaxing videos to create a relaxing environment; altered language used to reduce stressful trigger words for patients; provided an information booklet in a timely manner prior to the treatment to facilitate shared decision making; and increased staff presence in the interventional room to decrease feelings of isolation [50,58].

The prevalence of physical and psychosexual concerns following radiotherapy and the complexities of compliance with rehabilitative strategies, such as vaginal dilation, prompted researchers to counteract this with timely intervention strategies. Bergin *et al.* [69] and Schofield *et al.* [68] took a psychoeducational approach to improve coping, and reduce the psychological impact of treatment. The nurse-led consultations were included to provide tailored information at relevant timepoints throughout treatment to improve patient recall and involved elements such as radiotherapy department orientation to enhance patients’ sense of preparedness [68,69]. Peer support was a key component of the intervention to help patients make sense of their journey and provide social support [68,69]. Similarly, Jeffries *et al.* [52] developed a psychoeducational intervention, underpinned by a behaviour change model, involving groups of peers to normalise patient experience and overcome attitudinal barriers to care strategies. Kpoghomou *et al*. [70] introduced a programme in response to the lack of support patients are currently given regarding their sexual concerns, aiming to reduce side effects through supportive and educational measures.

Many of the nutritional interventions aimed to prevent or reduce the adverse gastrointestinal effects experienced by gynaecological patients, such as radiation enteritis, due to the impact on quality of life and treatment tolerability. Radiotherapy can disrupt the gut microbiota and cause intestinal inflammation and injury. A number of studies cited the positive effects of probiotics in patients with other inflammatory bowel conditions and theorised that probiotics may correct dysbiosis, improve or maintain intestinal barrier function, and/or offer beneficial immunomodulatory effects [81,82,88,93,97]. Whilst probiotic strains varied between studies, generally they were chosen due to safety and/or benefit in previous studies. Dietary changes and recommendations were often implemented to reduce or modify factors that may influence gastrointestinal toxicities. Bye *et al.* [110] theorised a low-fat, low-lactose diet would reduce diarrhoea during treatment by minimising faecal bile salt excretion and fluid accumulation within the intestinal lumen. However, they also highlighted that inadequate nutrition could impact healing so included compensatory measures and tailored advice to achieve sufficient energy intake and minimise weight loss. More recently, Soto-Lugo and colleagues [100] evaluated a low FODMAP diet to explore if gastrointestinal toxicity could be managed more effectively by modifying multiple factors rather than a single dietary element. Both Aredes *et al.* [103] and Medina-Jiménez *et al.* [95] aimed to reduce malnutrition and fat-free mass loss due to their association with treatment toxicity and poor outcomes. Aredes *et al.* [103] provided patients with nutritional risk omega-3 and nutritional formula supplements whereas Medina-Jiménez *et al.* [95] opted for a tailored nutritional intervention with individual counselling due to the reported benefits of this approach in other radiotherapy patient populations compared to standard recommendations.

#### What are the timings and duration of these interventions?.

**Fig 3:**
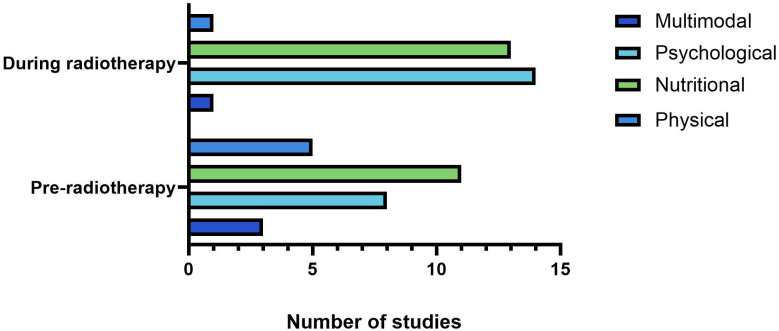
Point of initiation for unimodal and multimodal interventions.

Interventions were broadly divided between those beginning prior to radiotherapy (external-beam or brachytherapy) (n = 27) and those initiated at the point of or shortly after beginning radiotherapy (n = 29) (Fig 3). Point of intervention and duration was variable and difficult to determine for some studies due to ambiguous reporting. Most spanned the radiotherapy treatment period with some continuing for longer. The timings of individual studies are outlined in Tables 3-6, the duration of the interventions and number of sessions varied depending on factors such as treatment length (e.g., brachytherapy alone compared to chemoradiotherapy) and use of familiarisation periods.

#### What outcome measures are being reported within the literature?.

Outcome measures varied both across and within study categories. Quality of life was the most consistent outcome measured across studies. This was assessed by a variety of tools, used alone or in combination, including the European Organisation For Research And Treatment Of Cancer (EORTC) Core Quality of Life Questionnaire QLQ-C30 (n = 11) [27,31,37,39,53,86,88,94,100,103,108], EORTC QLQ-CX24 (cervical cancer module) (n = 3) [27,31,100], EORTC QLQ-EN24 (endometrial cancer module) (n = 1) [100], EORTC QLQ-36 (n = 1) [111], EuroQol (EQ)-5D-3L (n = 1) [103], EQ-5D-5L (n = 1) [28], Cuestionario de Calidad de Vida QL-CA-AFex (CCV) (n = 1) [60], Functional Assessment of Cancer Therapy (FACT) -General subscale (n = 5) [25,36,54,57,68], and FACT-Cervix (n = 1) [63].

Both ENABLE [25] and the ongoing RadBone trial [28] assess feasibility considering recruitment rate, attrition, and study eligibility. The ENABLE study utilised the Patient Generated-Subjective Global Assessment (PG-SGA), bioelectrical impedance, and accelerometery to assess nutritional and physical activity outcomes. Additionally, they included the Physical Activity Questionnaire-Short form and Physical Activity Assessment Inventory to measure patient-reported physical activity and self-efficacy levels respectively. Whilst the PG-SGA is used as part of the tailored nutritional intervention in Prehab4Cancer, the secondary outcome measures in RadBone centre around longitudinal changes including incidence of radiotherapy related insufficiency fractures measured by Magnetic Resonance Imaging (MRI); bone mineral density measured by Dual-Energy X-ray Absorptiometry (DEXA); biochemical markers of bone turnover; and fracture risk using the FRAX^®^ assessment tool [28]. Alongside the EQ-5D-5L, Grigoriadou *et al.* [28,33] are using the Patient-Reported Outcome Version of the Common Terminology Criteria for Adverse Events (PRO- CTCAE) and the Short Form Musculoskeletal Function Assessment to assess quality of life. Both Kaliamurthi *et al.* [27] and Andring *et al.* measured treatment related morbidity taking into consideration factors such as patient reported toxicity, hospital admissions and/or length of stay.

The distinct subgroups within the physical/exercise studies resulted in some shared outcomes. The studies evaluating prophylactic physiotherapy [34,39] both utilise patient-reported symptoms from the Gynecologic Cancer Lymphedema Questionnaire to identify risk and incidence of lower extremity lymphoedema. Pelvic floor strength was a primary outcome for the pelvic floor exercise studies, with Sacomori *et al.* [35] opting to measure pelvic floor strength using vaginal bidigital evaluation grading with the modified Oxford scale by a physical therapist and Jagdish *et al.* [38] using a perineometer. Both studies also used the International Consultation on Incontinence Questionnaire-Urinary Incontinence Short Form to assess urinary incontinence. The CTCAE was chosen by Tórtola-Navarro *et al.* [37] and Hauth *et al.* [36] to assess patient reported toxicity and adverse events. In the Tórtola-Navarro *et al.* [37] case study, the main outcome measure was feasibility of the programme determined by patient compliance but they were also interested in functional and exercise capacity changes using the Karnofsky index and both the Six Minute Walk Test and Five Times Sit to Stand Test.

In studies evaluating psychological interventions, common outcome measures included levels of psychological distress, anxiety and/or depression using tools such as: the Distress Thermometer (n = 1) [58]; Patient Reported Outcome Measurement Information System (PROMIS) Emotional-Distress Anxiety Short Form 4a (n = 1) [73]; the Hospital Anxiety and Depression Scale (n = 8) [43,46,53,58–60,68,72]; State-Trait Anxiety Inventory (n = 3) [56,62,67]; Six-item Spielberger State Anxiety Scale (n = 1) [43]; the Self-rating Anxiety Scale (n = 2) [63,65]; an 11-point anxiety numeric rating scale (n = 1) [71]; the Self-rating Depression scale (n = 1) [63]; the Beck Depression inventory (n = 1) [65]; and the Center for Epidemiological Studies Depression Scale (n = 1) [54]. Other outcomes included level of fatigue, assessed with the Chalder (n = 1) [61] Piper (n = 1) [63], or visual analogue (n = 1) [72] fatigue scales and pain levels using a numeric (n = 3) [61,62,71] or visual (n = 3) [43,56,72] rating scale. In addition to secondary outcomes of interest such as fatigue and pain levels, Texier and Meignant [72] are utilising a 10-item to perceived stress score to evaluate the impact of an educational physiotherapy yoga on perceived stress in cervical cancer patients undergoing brachytherapy. In the ongoing SISTER study, the primary outcome of interest is treatment completion rate measured by relative dose (expected vs actual dose received at six month follow-up) [73]. As a secondary measure, Oluloro *et al*. [73] are also evaluating the impact of the virtual support interventions on patient-reported social isolation using the Social Provisions Scale-24 and the PROMIS Short Form Social Isolation 4a.

For nutritional studies, incidence and severity of gastrointestinal toxicities were generally measured by versions of the CTCAE (n = 11) [82,83,86,88,91,93,96,99,100,103,112] and the Radiation Therapy Oncology Group (RTOG) criteria (n = 4) [84,95,99,105]. Additionally, some studies included the Bristol stool chart to monitor change in consistency or incidence of watery stools (n = 5) [80,81,85,86,88] and monitored the administration of intestinal regulators and/or anti-diarrhoeal agents during interventions (n = 7) [81,82,84,88,90,93,97]. A subset of studies (n = 4) [85,86,105,112] monitored faecal calprotectin levels as an indicator of intestinal inflammation whereas others monitored inflammation using markers such as c-reactive protein (n = 2) [80,105]. Body mass loss (n = 2) [100,110], body mass change (n = 4) [82,84,97,112] and change in body composition (n = 2) [95,103] were also frequently used to evaluate the impact of nutritional interventions. Like the ENABLE study, Aredes *et al.* [103] used the PG-SGA to assess nutritional status. However, they also utilised computed tomography (CT) images to evaluate changes body composition including skeletal muscle quantity and quality. Similarly, in an ongoing trial evaluating an immunonutrition intervention for individuals with nutritional risk, prevalence of malnutrition and sarcopenia are being assessed using a modified Global Leadership Initiative on Malnutrition criteria and the Skeletal Muscle Index respectively [108]. Other outcome measures for this study focus on survival including dose-limiting toxicity-free survival, two-year overall survival, and two-year progression free survival [108].

## Discussion

This scoping review identified 56 studies, spanning 66 years, evaluating unimodal or multimodal prehabilitation interventions for patients with gynaecological malignancies treated with radiotherapy. It is not entirely unexpected that cervical cancer was the most highly represented gynaecological cancer type across studies considering it is the 8^th^ most common cancer globally [116] and is more often treated with techniques such as brachytherapy than other tumour sites. Our findings indicate there are fewer studies focusing on multimodal prehabilitation programmes. Although, it is encouraging that ongoing trials like RadBone [28] are able to utilise established prehabilitation services and integrate them into a package tailored to radiotherapy patients. Despite many studies evaluating unimodal interventions, there is a clear imbalance between the categories, with physical exercise interventions being comparatively underrepresented.

The heterogeneity of studies in respect to intervention components, initiation, duration, delivery, outcome measures and the tools used to assess these, complicates future work in the form of a systematic review or meta-analysis to build on our findings. As the scope of this review is very broad, including a wide range of records from various countries spanning six decades, some variation was anticipated. Similar issues were identified by Saggu *et al.* [14] in their scoping review of prehabilitation interventions for gynaecological cancer patients receiving surgery. This is further compounded by inconsistent and incomplete reporting of interventions. Study heterogeneity and ambiguous reporting are frequently cited limitations of systematic reviews and meta-analyses in similar populations [1,15,117–120]. Promisingly, tools such as the TIDieR checklist are being used in more recent studies which has improved the completeness of reporting and replicability of interventions [25]. This is particularly important when considering the rationale of a complex intervention. Despite many authors expressing the need for an intervention to address the burden patients experience, few provided clear theory or rationale underpinning design and delivery. Prehabilitation is understudied in this population therefore comprehensive and transparent reporting is essential to facilitate future intervention development and inform changes in practice [121].

The total number of outcomes measured across studies and tools used to assess these was extensive, and incomplete reporting further impeded data charting. Despite the variation in outcome measures, there were a small number of validated instruments favoured by researchers. The EORTC QLQ-C30 is designed to measure health-related quality of life specifically in individuals with cancer [122]. The frequent use of this instrument is not surprising due to its length of circulation and availability in multiple languages. However, only a small proportion of studies used it alongside the recommended disease or symptom-specific modules. Similarly, the CTCAE provides an internationally recognised standard criteria for the reporting of adverse events in cancer therapy and clinical trials. Although more recently it has been acknowledged that additional use of PRO-CTCAE can provide greater insight into patient’s perception of toxicities which is beneficial to establishing tolerability of an intervention [123]. Several patient-reported outcome measures were also used across studies which highlights growing recognition of the importance of patient experience when developing and evaluating interventions [124]. The agreement of a core outcome set (COS) that are consistently measured by widely-accepted, validated tools will facilitate greater comparison between studies to inform future decisions regarding prehabilitation and this has also been recently acknowledged in surgical populations [125]. A project to agree standardised outcome measures for prehabilitation in cancer is currently being undertaken as part of the Core Outcome Measures in Effectiveness Trials (COMET) initiative [126]. However, it is important that consideration is given to how outcomes of interest and measures of efficacy may vary between different treatment populations [127].

Nutritional interventions in this population currently aim to address malnutrition and/or minimise GI toxicities. Despite advances in radiotherapy resulting in reduced morbidity and mortality, pelvic radiotherapy patients still experience acute and chronic GI toxicities which can impact quality of life and treatment tolerability [1]. Interruptions in treatment due toxicities and increase in overall treatment time impacts radiotherapy efficacy [1,128]. Therefore, nutritional support for gynaecological patients receiving radiotherapy is an important element of prehabilitation. Minimising the impact of radiation-induced dysbiosis and intestinal injury through the introduction of pre- and probiotics was a recurring concept in the literature, yet it is acknowledged more evidence is required to gauge the effectiveness of these interventions [1]. Additionally, malnutrition may occur due to changes in gastrointestinal absorption and digestive functioning. Nutritional interventions such as dietary changes are a proactive approach to addressing GI toxicity and improving nutritional status. However, the observed variation across studies highlights that there is not yet a clear approach for these interventions. Globally, differences in radiotherapy techniques, resources and cultural differences, including dietary preferences, may account for some of the observed variation in nutritional interventions.

The physical exercise interventions discussed in this review differ from those frequently used in prehabilitation for cancer surgery. Both the studies evaluating prophylactic physiotherapy for lymphoedema [34,39] and pelvic floor strengthening [35,38] exercises, highlight how prehabilitation can be adapted to address the unique challenges of a target population. There are several factors which may explain the comparative lack of studies evaluating physical exercise interventions. Prior to radiotherapy, patients have a CT scan to develop a highly conformal plan for treatment delivery. As such, subsequent changes in body mass are avoided as they can alter the planned dose to the target volumes and organs at risk [129]. Acute side effects such as vaginal mucositis, nausea; faecal and urinary urgency; and fatigue can present during radiotherapy treatment [1,2] which may influence patients’ desire and ability to exercise. Moreover, travelling to a radiotherapy department multiple times a week for several weeks restricts patients time to participate in additional activities, frequently cited as one of the major barriers to exercise [130]. In their case study, Tórtola-Navarro *et al.* [37] were able to deliver a highly tailored physical activity programme with consideration for factors such as delivery of chemotherapy which impacts patients’ general wellbeing and energy levels. In the pilot randomised controlled trial ENABLE [25], healthcare professionals worked with participants to develop a home-based physical activity programme with patient-centred nutritional and activity goals, yet some patients still expressed challenges engaging with the programme whilst navigating the side-effects of treatment.

It is well-established that gynaecological cancer and its subsequent treatment has a profound impact on psychological well-being [3,6,8]. The risk of traumatisation during brachytherapy further strengthens the need for psychological support in prehabilitation for these patients [6,7]. Studies have shown that gynaecological cancer survivors have unmet informational needs exacerbating existing psychological distress including increased anxiety and feelings of embarrassment [131–134]. Tailored information, provided in a timely manner, including involvement from both healthcare professionals and peers with lived experience can contribute to engagement, patient satisfaction and feelings of preparedness [135]. It is promising that a several studies identified this as an issue and incorporated elements of patient education, in diverse formats, to address this. The psychological interventions in this review ranged from brief interventions for relaxation during brachytherapy to more complex psychoeducational interventions addressing psychosexual concerns and providing social support. The multifactorial nature of psychological distress in gynaecological cancer patients necessitates a multifaceted approach, providing patients with relevant support at an appropriate time to minimise the impact of treatment-related burden.

Prehabilitation interventions are generally considered to be those delivered between diagnosis and the beginning of acute treatment [136]. This review included studies that introduce interventions both before and during the delivery of radiotherapy, with some spanning the pre-, intra- and post-treatment periods. Prehabilitation is an evolving concept and consideration must be given to each population’s unique needs [127]. As acknowledged by Flores *et al.* [22], the timing of intervention delivery is influenced by the nature of radiotherapy including its role within multimodal cancer care and delayed development of acute side effects. Therefore, including interventions implemented during radiotherapy is key to understanding prehabilitation in the context of radiotherapy, particularly if multiphasic prehabilitation becomes more prevalent in the future. In the multiphasic framework, prehabilitation is viewed as a health optimising strategy initiated at multiple time points after diagnosis [127]. Prehabilitation interventions are tailored to current and anticipated experiences throughout multimodal cancer care to minimise adverse effects and reduce treatment delay [127]. Prehabilitation for a gynaecological cancer patient prior to surgery may include aerobic exercise, resistance training, psychological support and pre-operative carbohydrate loading [137]. This patient may then go on to receive prehabilitation tailored for adjuvant radiotherapy such as prophylactic complex physiotherapy, nutritional interventions to reduce GI toxicity, pelvic floor strengthening, and psychosexual support including radiotherapy-specific education. If the patient is then scheduled for brachytherapy, they will receive further targeted interventions. As prehabilitation for non-surgical therapies continues to develop, we must examine the boundaries between prehabilitation and rehabilitation. If the end of prehabilitation continues to be defined by the beginning of treatment, there may be a gap for interventions during the treatment period that align with the principles of prehabilitation and go beyond standard supportive care.

The strengths of this scoping review lie in its comprehensive and inclusive approach to exploring a relatively understudied area. By including studies evaluating unimodal interventions, this review provides a holistic summary of the evidence base and offers invaluable insight into the components, timing and rationale of prehabilitation interventions in this population. Additionally, the broad inclusion criteria ensures that this review captures a wide range of evidence, including emerging research and recent innovations. This inclusive methodology strengthens the relevance and applicability of these findings to inform future research.

There are only a handful of studies evaluating multimodal prehabilitation in this population and these are limited to smaller studies and feasibility trials with limited published results. Whilst such studies provide valuable insight, larger randomised controlled trials are required in the future to strengthen the evidence base for prehabilitation in individuals with gynaecological malignancies undergoing radiotherapy. Future studies should prioritise complete and transparent reporting of interventions and adoption of a COS informed by multiple-stakeholders to be reported as a minimum across all cancer prehabilitation trials. This will reduce ambiguity and observed heterogeneity in the literature and facilitate data synthesis to establish efficacy and inform changes in practice and policy. Physical exercise interventions remain relatively unexplored and mixed-methods approaches, as was used in ENABLE [29], can provide valuable insight as to how we can adapt interventions to overcome perceived barriers to exercise in prehabilitation for patients undergoing (chemo)radiotherapy. Involving individuals with lived experience in the intervention development process, such as in the HAPPY [56], SISTER [71] and PeNTAGOn [66] trials, is an important element for ongoing research to ensure patients’ needs are being addressed in a way that is acceptable to them.

### Limitations

Despite an extensive literature search, it is possible that some studies may have been missed particularly as the term “prehabilitation” is not consistently used across the literature and only English language studies could be included due to limited resources. However, a significant amount of time was spent developing the search strategies and inclusion criteria, including extensive search terms, broad publication date ranges and information sources, to maximise the inclusion of relevant studies. It is outside the scope of this review to comment on the effectiveness of interventions and, as a risk of bias for studies was not performed, the results of this review are limited in terms of informing clinical guidance or policy. Nonetheless, this review captures the current landscape of prehabilitation in this population and highlights the gaps in current research.

## Conclusion

This review highlights the diverse research relating to prehabilitation for gynaecological cancer patients undergoing radiotherapy. Interventions to reduce functional impairment and address adverse patient experiences are not necessarily a new concept but there is growing consideration for the complexities of managing treatment and disease-related burden, with increased involvement of those with lived experiences during study development. Studies evaluating unimodal interventions are more prevalent and there remains gaps in knowledge and literature that need to be addressed. Physical exercise interventions are still relatively unexplored in this patient population and consideration must be given to the barriers to physical activity experienced by this patient group. The physical and psychological impacts of cancer diagnosis and treatment are closely entwined; therefore, further development of multimodal prehabilitation interventions to cohesively address these is an important area for future research. Larger, randomised controlled trials will be useful for establishing efficacy of prehabilitation for gynaecological cancer patients undergoing radiotherapy although researchers must recognise that a nuanced approach is required. Complete and transparent reporting of interventions, along with greater consistency in outcome measures, will allow for a more cohesive approach to prehabilitation for this patient population and facilitate change in both practice and policy.

## Supporting information

S1 Checklist PRISMA-ScR checklist. (DOCX)

S1 Table Search strategies for all databases (February 2024). (DOCX)

S2 Table Search strategies for all databases (October 2024). (DOCX)
