# Supplementary material for: Exploring prehabilitation interventions for patients with gynaecological cancer undergoing radiotherapy: A scoping review
Source: PLoS One. 2025 Mar 13;20(3):e0319518. doi: 10.1371/journal.pone.0319518 (PMC11906083; doi:10.1371/journal.pone.0319518)
Supplement: S2 Table — (DOCX) [file pone.0319518.s003.docx]

**Search strategies**

| S3 Table 1. Search strategies for grey literature repositories | | | |
| --- | --- | --- | --- |
| Grey literature repository | Search terms | | No. of results |
| Google Scholar^A^  14/10/24 | (“Gynecology” OR “Gynaecology” OR “Gynecological” OR “Gynaecological” OR “Ovary” OR “Ovarian” OR “Uterus” OR “Uterine” OR “Cervix” OR “Cervical” OR “Endometrium” OR “Endometrial” OR “Vagina” OR “Vaginal” OR “Vulva” OR “Vulval” OR “Vulvar” OR “Fallopian”) (Cancer OR tumour OR tumor OR neoplasm) (“Prehabilitation” OR “Prehab” OR “Pre-hab” OR “Pre-habilitation”) | | 11 |
|  | (“Gynecology” OR “Gynaecology” OR “Gynecological” OR “Gynaecological” OR “Ovary” OR “Ovarian” OR “Uterus” OR “Uterine” OR “Cervix” OR “Cervical” OR “Endometrium” OR “Endometrial” OR “Vagina” OR “Vaginal” OR “Vulva” OR “Vulval” OR “Vulvar” OR “Fallopian”) (Cancer OR tumour OR tumor OR neoplasm) (“exercise” OR “exercises” OR “exercising”) | | 19 |
|  | (“Gynecology” OR “Gynaecology” OR “Gynecological” OR “Gynaecological” OR “Ovary” OR “Ovarian” OR “Uterus” OR “Uterine” OR “Cervix” OR “Cervical” OR “Endometrium” OR “Endometrial” OR “Vagina” OR “Vaginal” OR “Vulva” OR “Vulval” OR “Vulvar” OR “Fallopian”) (Cancer OR tumour OR tumor OR neoplasm) (“Nutrition” OR “nutritional” OR “diet” OR “dietary”) | | 65 |
|  | (“Gynecology” OR “Gynaecology” OR “Gynecological” OR “Gynaecological” OR “Ovary” OR “Ovarian” OR “Uterus” OR “Uterine” OR “Cervix” OR “Cervical” OR “Endometrium” OR “Endometrial” OR “Vagina” OR “Vaginal” OR “Vulva” OR “Vulval” OR “Vulvar” OR “Fallopian”) (Cancer OR tumour OR tumor OR neoplasm) (“psychological” OR “psychosocial” OR “counseling” OR “counselling”) | | 62 |
| Trip Pro Medical  15/10/24 | #1 | (title:gynecology OR title:gynaecology OR title:gynecological OR title:gynaecological OR title:ovary OR title:ovarian OR title:uterus OR title:uterine OR title:cervix OR title:cervical OR title:endometrium OR title:endometrial OR title:vagina OR title:vaginal OR title:vulva OR title:vulval OR title:vulvar OR title:fallopian) | 107,525 |
|  | #2 | (title:cancer OR title:tumour OR title:tumor OR title:neoplasm) | 462,181 |
|  | #3 | (title:prehabilitation OR title:prehab OR title:pre-hab OR title:pre-habilitation OR title:exercise OR title:nutrition OR title:diet OR title:nutritional OR title:dietary OR title:psychological OR title:psychosocial OR title:counseling OR title:counselling) | 242,064 |
|  | #4 | #1 AND #2 AND #3 | 618 |
|  | #5 | (radiotherapy OR "radiation therapy" OR brachytherapy OR chemoradiotherapy) | 135,546 |
|  | #6 | #4 AND #5 | 37^B^ |
| Overton.io  15/10/24 | (Gyne* OR Gynae* OR Ovar* OR Uter* OR Cervi* OR Endomet* OR Vagina* OR Vulva* OR Fallopian) AND (Cancer OR tumo* OR neoplasm) AND (Prehab* OR Pre-hab OR Pre-habilitation OR exercis* OR nutrition* OR diet* OR psych* OR counsel*) AND (Radiotherapy OR “Radiation therapy” OR Brachytherapy OR Chemoradiotherapy) ^C^ | | 2 |
|  | title:(Cancer OR tumo* OR neoplasm) AND (exercis* OR "physical activity") ^D^ | | 5 |
|  | title:(Cancer OR tumo* OR neoplasm) AND (nutrition* OR diet*) ^D^ | | 21 |
|  | title:(Cancer OR tumo* OR neoplasm) AND (psych* OR counsel*) ^D^ | | 11 |
|  | title:(radiotherapy OR “radiation therapy” OR brachytherapy OR chemoradiotherapy) AND (exercis* OR "physical activity") ^D^ | | 0 |
|  | title:(radiotherapy OR “radiation therapy” OR brachytherapy OR chemoradiotherapy) AND (nutrition* OR diet*) ^D^ | | 0 |
|  | title:(radiotherapy OR “radiation therapy” OR brachytherapy OR chemoradiotherapy) AND (psych* OR counsel*) ^D^ | | 0 |
|  | title:(prehab* OR Pre-hab OR Pre-habilitation) ^D^ | | 1 |

^A^ Google Scholar: The search terms were entered as written into the “with all of the words” field of the advanced search interface with “in title of the article” selected.

^B^ Trip Pro Medical: Limited interface to limit search by publication date. Ordered results by publication and exported the 4 results that had been added since the last literature search conducted in February 2024.

^C^ Overton.io: This search was conducted in the scholarly articles section.

^D^ Overton.io: Overton.io: This search was conducted in the policy documents section.

| S3 Table 2. Search strategy for CINAHL 9/10/24 | | | |
| --- | --- | --- | --- |
| Search no. | Search term | No. of results | New results |
| S1 | TI ( cancer* OR neoplas* OR carcinoma* OR malignan* OR tumo#r* OR oncolog* ) OR AB ( cancer* OR neoplas* OR carcinoma* OR malignan* OR tumo#r* OR oncolog* ) | 734,907 | 29,825 |
| S2 | (MH "Neoplasms") | 93,940 | 1,953 |
| S3 | S1 OR S2 | 753,557 | 30,161 |
| S4 | TI ( Gyn#ecolog* OR ovar* OR uter* OR womb OR endometr* OR vagin* OR cervi* OR vulva* OR fallopian ) OR AB ( Gyn#ecolog* OR ovar* OR uter* OR womb OR endometr* OR vagin* OR cervi* OR vulva* OR fallopian ) | 188,001 | 6,428 |
| S5 | (MH "Gynecology") | 5, 081 | 106 |
| S6 | (MH "Genital Diseases, Female") OR (MH "Uterine Diseases") OR (MH "Vaginal Diseases") OR (MH "Vulvar Diseases") OR (MH "Adnexal Diseases+") | 31,900 | 605 |
| S7 | S4 OR S5 OR S6 | 197,620 | 6,623 |
| S8 | S3 AND S7 | 62,849 | 2,331 |
| S9 | (MH "Genital Neoplasms, Female") OR (MH "Ovarian Neoplasms+") OR (MH "Uterine Neoplasms") OR (MH "Cervix Neoplasms+") OR (MH "Endometrial Neoplasms") OR (MH "Vaginal Neoplasms") OR (MH "Vulvar Neoplasms") | 47,141 | 922 |
| S10 | S8 OR S9 | 74,491 | 2,508 |
| S11 | TI ( Prehab* OR pre-hab* OR pre-rehab* ) OR AB ( Prehab* OR pre-hab* OR pre-rehab* ) | 823 | 105 |
| S12 | (MH “Prehabilitation”) | 377 | 75 |
| S13 | S11 OR S12 | 972 | 129 |
| S14 | S10 AND S13 | 21 | 6 |
| S15 | (MH "Radiotherapy") OR (MH "Brachytherapy") OR (MH "Chemoradiotherapy+") OR (MH "Radiotherapy, Conformal") | 30,992 | 693 |
| S16 | TI ( Radiotherapy OR “Radiation therapy” OR EBRT OR Brachytherapy OR Chemoradiotherapy ) OR AB ( Radiotherapy OR “Radiation therapy” OR EBRT OR Brachytherapy OR Chemoradiotherapy ) | 69,158 | 3,119 |
| S17 | S15 OR S16 | 80,354 | 3,339 |
| S18 | S14 AND S17 | 3 | 1 |
| S19 | (MH "Exercise") OR (MH "Therapeutic Exercise") OR (MH "Aerobic Exercises+") OR (MH "Running+") OR (MH "Walking+") OR (MH "Anaerobic Exercises") OR (MH "Abdominal Exercises") OR (MH "Core Exercises") OR (MH "Group Exercise") OR (MH "High-Intensity Interval Training") OR (MH "Physical Activity") OR (MH "Endurance Training") OR (MH "Conditioning, Cardiopulmonary") OR (MH "Exercise Test, Cardiopulmonary") OR (MH "Exercise Test") OR (MH "Exercise Tolerance+") OR (MH "Weight Lifting") OR (MH "Muscle Strengthening+") OR (MH "Callisthenics") OR (MH "Plyometrics") OR (MH "Kegel Exercises") OR (MH "Pilates") OR (MH "Yoga") OR (MH "Stretching") OR (MH "Movement") OR (MH "Swimming") OR (MH "Cycling") | 255,395 | 7,427 |
| S20 | TI ( Exercise OR “aerobics” OR dancing OR dance OR run* OR jog* OR walk* OR “high-intensity interval” OR “high intensity interval” OR HIIT OR “Low-intensity steady state” OR “Low intensity steady state” OR LISS OR “physical activity” OR endurance OR training OR fitness OR weightlifting OR “weight lifting” OR cal#isthenics OR plyometric OR kegel OR “pelvic floor” OR dilator* OR pilates OR yoga OR stretch* OR flexibility OR swimming OR cycling ) OR AB (Exercise OR “aerobics” OR dancing OR dance OR run* OR jog* OR walk* OR “high-intensity interval” OR “high intensity interval” OR HIIT OR “Low-intensity steady state” OR “Low intensity steady state” OR LISS OR “physical activity” OR endurance OR training OR fitness OR weightlifting OR “weight lifting” OR cal#isthenics OR plyometric OR kegel OR “pelvic floor” OR dilator* OR pilates OR yoga OR stretch* OR flexibility OR swimming OR cycling ) | 523,658 | 17,992 |
| S21 | S19 OR S20 | 612,655 | 20,281 |
| S22 | S10 AND S17 AND S21 | 209 | 11 |
| S23 | (MH "Psychosocial Intervention") OR (MH "Support, Psychosocial") OR (MH "Rehabilitation, Psychosocial”) OR (MH "Psychological Well-Being”) OR (MH "Psychotherapy") OR (MH "Psychotherapy, Brief+") OR (MH "Psychotherapy, Group") OR (MH "Counseling") OR (MH "Couples Counseling") OR (MH "Sexual Counseling") OR (MH "Mental Health Counseling") OR (MH "Cognitive Therapy") OR (MH "Dialectical Behavior Therapy") OR (MH "Cognitive Restructuring") OR (MH "Desensitization, Psychologic+") OR (MH "Mind Body Techniques") OR (MH "Mindfulness+") OR (MH "Relaxation Techniques") OR (MH "Guided Imagery") OR (MH "Meditation") OR (MH "Hypnosis+") OR (MH "Stress Management") OR (MH "Music Therapy") OR (MH "Holistic Care") OR (MH "Reiki") | 254,193 | 12,115 |
| S24 | TI (“Psycho* intervention*” OR “psycho* support” OR “well-being” OR “wellbeing” OR psychotherap* OR counsel#ing OR “support group” OR “cognitive therap*” OR “cognitive behavio#r* therapy” OR CBT OR “dialectical behavio#r therapy” OR “cognitive restructuring” OR “eye movement desensitisation and reprogramming” OR “eye movement desensitization and reprogramming” OR EMDR OR mindfulness OR relaxation OR meditation OR hypnotherap* OR “stress management” OR “music therap*” OR “holistic need* assessment” OR HNA OR reiki ) OR AB (“Psycho* intervention*” OR “psycho* support” OR “well-being” OR “wellbeing” OR psychotherap* OR counsel#ing OR “support group” OR “cognitive therap*” OR “cognitive behavio#r* therapy” OR CBT OR “dialectical behavio#r therapy” OR “cognitive restructuring” OR “eye movement desensitisation and reprogramming” OR “eye movement desensitization and reprogramming” OR EMDR OR mindfulness OR relaxation OR meditation OR hypnotherap* OR “stress management” OR “music therap*” OR “holistic need* assessment” OR HNA OR reiki ) | 202,267 | 8,790 |
| S25 | S23 OR S24 | 370,361 | 15,957 |
| S26 | S10 AND S17 AND S25 | 155 | 11 |
| S27 | (MH "Nutrition Services") OR (MH "Nutritional Counseling") OR (MH "Nutrition Education") OR (MH "Nutritional Support") OR (MH "Dietary Supplementation") OR (MH "Home Nutritional Support") OR (MH "Nutritional Assessment") OR (MH "Telenutrition") OR (MH "Dietary Supplements") OR (MH "Probiotics+") OR (MH "Dietary Fiber") OR (MH "Whey Proteins") OR (MH "Plant Proteins") OR (MH "Food, Fortified") OR (MH "Nutritional Support Team") OR (MH "Diet") OR (MH "Antioxidants") | 173,097 | 5,061 |
| S28 | TI ( Nutrition* OR diet* OR fiber OR fibre OR “omega-3” OR “omega 3” OR “omega3” OR antioxidant* OR “malnutrition universal screening tool” OR “MUST” OR “subjective global assessment” OR SGA OR PG-SGA OR “short nutrition assessment questionnaire” OR SNAQ OR “health package” ) OR AB ( Nutrition* OR diet* OR fiber OR fibre OR “omega-3” OR “omega 3” OR “omega3” OR antioxidant* OR “malnutrition universal screening tool” OR “MUST” OR “subjective global assessment” OR SGA OR PG-SGA OR “short nutrition assessment questionnaire” OR SNAQ OR “health package”) | 404,436 | 12,423 |
| S29 | S27 OR S28 | 480,878 | 14,241 |
| S30 | S10 AND S17 AND S29 | 206 | 8 |
| S31 | S18 OR S22 OR S26 OR S30 | 522 | 26 |
| S32 | S31 (Limiters - English Language; Language: English) | 507 | 22 |

| S3 Table 3. Search strategy for MEDLINE 14/10/2024 | | | |
| --- | --- | --- | --- |
| Search no. | Search term | No. of results | New results |
| S1 | TI ( cancer* OR neoplas* OR carcinoma* OR malignan* OR tumo#r* OR oncolog* ) OR AB ( cancer* OR neoplas* OR carcinoma* OR malignan* OR tumo#r* OR oncolog* ) | 4,128,862 | 165,472 |
| S2 | (MH "Neoplasms") | 513,796 | 15,244 |
| S3 | S1 OR S2 | 4,217,757 | 165,814 |
| S4 | TI ( Gyn#ecolog* OR ovar* OR uter* OR womb OR endometr* OR vagina* OR cervi* OR vulva* OR fallopian ) OR AB ( Gyn#ecolog* OR ovar* OR uter* OR womb OR endometr* OR vagina* OR cervi* OR vulva* OR fallopian ) | 1,002,920 | 31,534 |
| S5 | (MH "Gynecology") | 20,978 | 363 |
| S6 | (MH "Genital Diseases, Female") OR (MH "Ovarian Diseases") OR (MH "Uterine Diseases") OR (MH "Uterine Cervical Diseases") OR (MH "Vulvar Diseases") OR (MH "Vaginal Diseases") OR (MH "Adnexal Diseases") OR (MH "Fallopian Tube Diseases") | 49,158 | 358 |
| S7 | S4 OR S5 OR S6 | 1,023,197 | 31,638 |
| S8 | S3 AND S7 | 348,463 | 12,672 |
| S9 | (MH "Genital Neoplasms, Female") OR (MH "Vulvar Neoplasms") OR (MH "Vaginal Neoplasms") OR (MH "Uterine Neoplasms+") OR (MH "Fallopian Tube Neoplasms") OR (MH "Ovarian Neoplasms") OR (MH "Carcinoma, Ovarian Epithelial") | 259,686 | 5,354 |
| S10 | S8 OR S9 | 412,590 | 13,105 |
| S11 | TI ( Prehab* OR pre-hab* OR pre-rehab* ) OR AB ( Prehab* OR pre-hab* OR pre-rehab* ) | 1,829 | 341 |
| S12 | S10 AND S11 | 51 | 10 |
| S13 | (MH "Radiotherapy") OR (MH "Brachytherapy") OR (MH "Chemoradiotherapy+") OR (MH "Radiotherapy, Conformal") | 92,503 | 1,377 |
| S14 | TI ( Radiotherapy OR “Radiation therapy” OR EBRT OR Brachytherapy OR Chemoradiotherapy ) OR AB ( Radiotherapy OR “Radiation therapy” OR EBRT OR Brachytherapy OR Chemoradiotherapy ) | 306,027 | 12,217 |
| S15 | S13 OR S14 | 338,120 | 12,380 |
| S16 | S12 AND S15 | 8 | 0 |
| S17 | (MH "Exercise") OR (MH "Exercise Therapy") OR (MH "Dancing") OR (MH "Dance Therapy") OR (MH "Aquatic Therapy") OR (MH "Running") OR (MH "Jogging") OR (MH "Walking") OR (MH "High-Intensity Interval Training") OR (MH "Circuit-Based Exercise") OR (MH "Endurance Training") OR (MH "Exercise Test+") OR (MH "Exercise Tolerance") OR (MH "Weight Lifting") OR (MH "Resistance Training") OR (MH "Plyometric Exercise") OR (MH "Exercise Movement Techniques") OR (MH "Yoga") OR (MH "Muscle Stretching Exercises") OR (MH "Swimming") OR (MH "Bicycling") | 344,528 | 10,560 |
| S18 | TI ( Exercise OR “aerobics” OR dancing OR dance OR run* OR jog* OR walk* OR “high-intensity interval” OR “high intensity interval” OR HIIT OR “Low-intensity steady state” OR “Low intensity steady state” OR LISS OR “physical activity” OR endurance OR training OR OR fitness OR weightlifting OR “weight lifting” OR cal#isthenics OR plyometric OR kegel OR “pelvic floor” OR dilators OR pilates OR yoga OR stretch* OR flexibility OR swimming OR cycling ) OR AB ( Exercise OR “aerobics” OR dancing OR dance OR run* OR jog* OR walk* OR “high-intensity interval” OR “high intensity interval” OR HIIT OR “Low-intensity steady state” OR “Low intensity steady state” OR LISS OR “physical activity” OR endurance OR training OR conditioning OR fitness OR weightlifting OR “weight lifting” OR cal#isthenics OR plyometric OR kegel OR “pelvic floor” OR dilators OR pilates OR yoga OR stretch* OR flexibility OR swimming OR cycling ) | 1,122, 820 | 53,713 |
| S19 | S17 OR S18 | 1,196,375 | 54,589 |
| S20 | S10 AND S15 AND S19 | 361 | 18 |
| S21 | (MH "Psychosocial Intervention") OR (MH "Psychosocial Support Systems") OR (MH "Psychiatric Rehabilitation") OR (MH "Psychological Well-Being") OR (MH "Psychotherapy") OR (MH "Psychotherapy, Brief") OR (MH "Schema Therapy") OR (MH "Psychotherapy, Group") OR (MH "Counseling") OR (MH "Couples Therapy") OR (MH "Sex Counseling") OR (MH "Cognitive Behavioral Therapy") OR (MH "Dialectical Behavior Therapy") OR (MH "Cognitive Restructuring") OR (MH "Desensitization, Psychologic+") OR (MH "Mind-Body Therapies") OR (MH "Mindfulness") OR (MH "Relaxation Therapy") OR (MH "Relaxation") OR (MH "Imagery, Psychotherapy") OR (MH "Meditation") OR (MH "Hypnosis+") OR (MH "Music Therapy") OR (MH "Holistic Health") OR (MH "Therapeutic Touch") | 186,253 | 3,865 |
| S22 | TI ( "Psycho* intervention" OR "Psycho* interventions" OR "psycho* support" OR "well-being" OR "wellbeing" OR psychotherap* OR counsel#ing OR "support group" OR "support groups" OR "cognitive therap*" OR "cognitive behavio#r* therapy" OR CBT OR "dialectical behavio#r therapy" OR "cognitive restructuring" OR "eye movement desensitisation and reprogramming" OR “eye movement desensitization and reprogramming” OR mindfulness OR relaxation OR meditation OR hypnotherap* OR "stress management" OR "music therap*" OR "holistic need* assessment" OR HNA OR reiki ) OR AB ( "Psycho* intervention" OR "Psycho* interventions" OR "psycho* support" OR "well-being" OR "wellbeing" OR psychotherap* OR counsel#ing OR "support group" OR "support groups" OR "cognitive therap*" OR "cognitive behavio#r* therapy" OR CBT OR "dialectical behavio#r therapy" OR "cognitive restructuring" OR "eye movement desensiti?ation and reprogramming" OR mindfulness OR relaxation OR meditation OR hypnotherap* OR "stress management" OR "music therap*" OR "holistic need* assessment" OR HNA OR reiki ) | 523,446 | 31,468 |
| S23 | S21 OR S22 | 619,977 | 32,402 |
| S24 | S10 AND S15 AND S23 | 316 | 21 |
| S25 | (MH "Nutrition Therapy") OR (MH "Diet Therapy") OR (MH "Nutritional Support") OR (MH "Dietary Supplements") OR (MH "Dietary Fiber") OR (MH "Probiotics+") OR (MH "Prebiotics") OR (MH "Whey Proteins") OR (MH "Plant Proteins, Dietary") OR (MH "Food, Fortified") OR (MH "Antioxidants") OR (MH "Nutrition Assessment") | 319,135 | 12,719 |
| S26 | TI ( Nutrition* OR diet* OR fiber OR fibre OR “omega-3” OR “omega 3” OR “omega3” OR antioxidant* OR “malnutrition universal screening tool” OR “MUST” OR “subjective global assessment” OR SGA OR PG-SGA OR “short nutrition assessment questionnaire” OR SNAQ OR “health package” ) OR AB ( Nutrition* OR diet* OR fiber OR fibre OR “omega-3” OR “omega 3” OR “omega3” OR antioxidant* OR “malnutrition universal screening tool” OR “MUST” OR “subjective global assessment” OR SGA OR PG-SGA OR “short nutrition assessment questionnaire” OR SNAQ OR “health package” ) | 2,069,212 | 87,919 |
| S27 | S25 OR S26 | 2,168,592 | 90,853 |
| S28 | S10 AND S15 AND S27 | 1,018 | 37 |
| S29 | S20 OR S24 OR S28 | 1,691 | 72 |
| S30 | S29 (Limiters - English Language; Language: English) | 1,429 | 69 |

| S3 Table 4. Search strategy for PsycInfo 9/10/24 | | | |
| --- | --- | --- | --- |
| Search no. | Search term | No. of results | New results |
| S1 | TI ( cancer* OR neoplas* OR carcinoma* OR malignan* OR tumo#r* OR oncolog* ) OR AB ( cancer* OR neoplas* OR carcinoma* OR malignan* OR tumo#r* OR oncolog* ) | 96,765 | 1,519 |
| S2 | DE "Neoplasms" | 48,185 | 635 |
| S3 | S1 OR S2 | 99,148 | 1,534 |
| S4 | TI ( Gyn#ecolog* OR ovar* OR uter* OR womb OR endometr* OR vagina* OR cervi* OR vulva* OR fallopian ) OR AB ( Gyn#ecolog* OR ovar* OR uter* OR womb OR endometr* OR vagina* OR cervi* OR vulva* OR fallopian ) | 34,857 | 551 |
| S5 | DE "Gynecology" | 1,350 | 15 |
| S6 | DE "Gynecological Disorders" OR DE "Genital Disorders" | 1,865 | 29 |
| S7 | S4 OR S5 OR S6 | 35,993 | 556 |
| S8 | S3 AND S7 | 6,488 | 112 |
| S9 | TI ( Prehab* OR pre-hab* OR pre-rehab* ) OR AB ( Prehab* OR pre-hab* OR pre-rehab* ) | 108 | 5 |
| S10 | S8 AND S9 | 5 | 0 |
| S11 | TI ( Radiotherapy OR “Radiation therapy” OR EBRT OR Brachytherapy OR Chemoradiotherapy ) OR AB ( Radiotherapy OR “Radiation therapy” OR EBRT OR Brachytherapy OR Chemoradiotherapy ) | 3,063 | 47 |
| S12 | S10 AND S11 | 0 | 0 |
| S13 | DE "Exercise" OR DE "Exercise Therapy" OR DE "Aerobic Exercise" OR DE "Running" OR DE "Walking" OR DE "Physical Activity" OR DE "Physical Endurance" OR DE "Sport and Exercise Measures" OR DE "Weightlifting" OR DE "Yoga" OR DE "Movement Therapy" OR DE "Swimming" OR DE "Cycling" | 76,756 | 1,579 |
| S14 | TI ( Exercise OR “aerobics” OR dancing OR dance OR run* OR jog* OR walk* OR “high-intensity interval” OR “high intensity interval” OR HIIT OR “Low-intensity steady state” OR “Low intensity steady state” OR LISS OR “physical activity” OR endurance OR training OR fitness OR weightlifting OR “weight lifting” OR cal#isthenics OR plyometric OR kegel OR “pelvic floor” OR dilator* OR pilates OR yoga OR stretch* OR flexibility OR swimming OR cycling ) OR AB ( Exercise OR “aerobics” OR dancing OR dance OR run* OR jog* OR walk* OR “high-intensity interval” OR “high intensity interval” OR HIIT OR “Low-intensity steady state” OR “Low intensity steady state” OR LISS OR “physical activity” OR endurance OR training OR fitness OR weightlifting OR “weight lifting” OR cal#isthenics OR plyometric OR kegel OR “pelvic floor” OR dilator* OR pilates OR yoga OR stretch* OR flexibility OR swimming OR cycling ) | 534,975 | 9,081 |
| S15 | S13 OR S14 | 543,521 | 9,186 |
| S16 | S8 AND S11 AND S15 | 19 | 0 |
| S17 | DE "Psychosocial Interventions" OR DE "Psychosocial Rehabilitation" OR DE "Psychoeducation" OR DE "Well Being" OR DE "Psychotherapy" OR DE "Brief Psychotherapy" OR DE "Group Psychotherapy" OR DE "Group Counseling" OR DE "Psychotherapeutic Counseling" OR DE "Counseling" OR DE "Online Therapy" OR DE "Couples Therapy" OR DE "Sex Therapy" OR DE "Compassion Focused Therapy” OR DE "Cognitive Therapy" OR DE "Cognitive Behavior Therapy" OR DE "Dialectical Behavior Therapy" OR DE "Cognitive Processing Therapy" OR DE "Systematic Desensitization Therapy" OR DE "Eye Movement Desensitization Therapy" OR DE "Mind Body Therapy" OR DE "Mindfulness" OR DE "Mindfulness-Based Interventions" OR DE "Mindfulness-Based Stress Reduction" OR DE "Mindfulness-Based Cognitive Therapy" OR DE "Mindfulness Meditation" OR DE "Relaxation Therapy" OR DE "Guided Imagery" OR DE "Meditation" OR DE "Hypnotherapy" OR DE "Anxiety Management" OR DE "Music Therapy" OR DE "Holistic Health" | 290,499 | 4,822 |
| S18 | TI ( “Psycho* intervention*” OR “psycho* support” OR “well-being” OR “wellbeing” OR psychotherap* OR counsel#ing OR “support group” OR “cognitive therap*” OR “cognitive behavio#r* therapy” OR CBT OR “dialectical behavio#r therapy” OR “cognitive restructuring” OR “eye movement desensitisation and reprogramming” OR “eye movement desensitization and reprogramming” OR EMDR OR mindfulness OR relaxation OR meditation OR hypnotherap* OR “stress management” OR “music therap*” OR “holistic need* assessment” OR HNA OR reiki ) OR AB ( “Psycho* intervention*” OR “psycho* support” OR “well-being” OR “wellbeing” OR psychotherap* OR counsel#ing OR “support group” OR “cognitive therap*” OR “cognitive behavio#r* therapy” OR CBT OR “dialectical behavio#r therapy” OR “cognitive restructuring” OR “eye movement desensitisation and reprogramming” OR “eye movement desensitization and reprogramming” OR EMDR OR mindfulness OR relaxation OR meditation OR hypnotherap* OR “stress management” OR “music therap*” OR “holistic need* assessment” OR HNA OR reiki ) | 416,923 | 8,903 |
| S19 | S17 OR S18 | 496,976 | 9,651 |
| S20 | S8 AND S11 AND S19 | 20 | 2 |
| S21 | DE "Nutrition" OR DE "Dietary Treatment" OR DE "Dietary Supplements" OR DE "Diets" OR DE "Antioxidants" | 33,049 | 551 |
| S22 | TI ( Nutrition* OR diet* OR fiber OR fibre OR “omega-3” OR “omega 3” OR “omega3” OR antioxidant* OR “malnutrition universal screening tool” OR “MUST” OR “subjective global assessment” OR SGA OR PG-SGA OR “short nutrition assessment questionnaire” OR SNAQ OR “health package” ) OR AB ( Nutrition* OR diet* OR fiber OR fibre OR “omega-3” OR “omega 3” OR “omega3” OR antioxidant* OR “malnutrition universal screening tool” OR “MUST” OR “subjective global assessment” OR SGA OR PG-SGA OR “short nutrition assessment questionnaire” OR SNAQ OR “health package” ) | 264,893 | 3,441 |
| S23 | S21 OR S22 | 269,332 | 3,490 |
| S24 | S8 AND S11 AND S23 | 10 | 0 |
| S25 | S10 OR S16 OR S20 OR S24 | 42 | 2 |
| S26 | S25 (Limiters - English language; Language: English) | 40 | 2 |

| S3 Table 5. Search strategy for EMBASE 14/10/2024 | | | |
| --- | --- | --- | --- |
| Search no. | Search term | No. of results | New results |
| 1 | (cancer* OR neoplas* OR carcinoma* OR malignan* OR tumo?r* OR oncolog*).ti,ab. | 5,534,069 | 5,760,592 |
| 2 | Neoplasm.sh. | 447,602 | 452,125 |
| 3 | 1 OR 2 | 5,571,121 | 5,798,021 |
| 4 | (Gyn?ecolog* or ovar* or uter* or womb or endometr* or vagina* or cervix or cervical cancer or vulva* or fallopian).ti,ab. | 1,062,596 | 1,341,660 |
| 5 | (Gynecology or Gynecologic disease or Adnexa disease or Ovary disease or Uterus disease or Uterine body disease or Uterine cervix disease or Endometrial disease or Vagina disease or Vulva disease).sh. | 86,969 | 89,764 |
| 6 | 4 OR 5 | 1,085,573 | 1,363,512 |
| 7 | 3 AND 6 | 424,275 | 501,479 |
| 8 | (Female genital tract tumor or Ovary tumor or Fallopian tube tumor or "adnexal tumor (gynecologic)" or uterus tumor or uterine cervix tumor or endometrium tumor or vagina tumor or vulva tumor).sh. | 69,200 | 71,138 |
| 9 | 7 OR 8 | 444,242 | 520,004 |
| 10 | (Prehab* OR pre hab* OR pre rehab*).ti,ab. | 2,925 | 3,391 |
| 11 | 9 AND 10 | 94 | 124 |
| 12 | (radiotherapy or cancer radiotherapy or vaginal brachytherapy or brachytherapy or chemoradiotherapy or adjuvant chemoradiotherapy or conformal radiotherapy or external beam radiotherapy).sh. | 495,319 | 515,828 |
| 13 | (Radiotherapy OR Radiation therapy OR EBRT OR Brachytherapy OR Chemoradiotherapy).ti,ab. | 473,616 | 492,460 |
| 14 | 12 OR 13 | 654,088 | 680,640 |
| 15 | 11 AND 14 | 11 | 17 |
| 16 | (Exercise or aerobic exercise or kinesiotherapy or running or jogging or walking or anaerobic exercise or low intensity exercise or high intensity exercise or high intensity interval training or endurance training or cardiopulmonary exercise test or exercise test or weight lifting or exercise tolerance or physical capacity or isokinetic exercise or isometric exercise or isotonic exercise or calisthenics or plyometrics or pelvic floor muscle training or pilates or yoga or stretching or stretching exercise or swimming or cycling).sh. | 642,037 | 670,926 |
| 17 | (Exercise OR aerobics OR dance OR dancing OR run* OR jog* OR walk* OR high intensity interval OR HIIT OR Low intensity steady state OR LISS OR physical activity OR endurance OR training OR fitness OR weightlifting OR weight lifting OR cal?isthenics OR plyometric OR kegel OR pelvic floor OR dilator* OR pilates OR yoga OR stretch* OR flexibility OR swimming OR cycling).ti,ab. | 2,052,971 | 2,149,861 |
| 18 | 16 OR 17 | 2,253,114 | 2,358,280 |
| 19 | 9 AND 14 AND 18 | 1,651 | 2,007 |
| 20 | (psychosocial intervention or psychosocial care or psychological care or psychosocial rehabilitation or psychological well-being or psychotherapy or short term psychotherapy or schema therapy or group therapy or counseling or couple therapy or sexual counseling or psychological counseling or cognitive therapy or cognitive behavioral therapy or dialectical behavior therapy or cognitive restructuring or "eye movement desensitization and reprocessing" or virtual reality exposure therapy or alternative medicine or mindfulness-based cognitive therapy or mindfulness-based stress reduction or mindfulness or mindfulness meditation or relaxation training or guided imagery or meditation or hypnosis or stress management or music therapy or holistic care).sh. | 409,588 | 427,804 |
| 21 | (Psycho* intervention* OR psycho* support OR well-being OR wellbeing OR psychotherap* OR counsel?ing OR support group OR cognitive therap* OR cognitive behavio?r* therapy OR CBT OR dialectical behavio?r therapy OR cognitive restructuring OR eye movement desensiti?ation reprogramming OR EMDR OR mindfulness OR relaxation OR meditation OR hypnotherap* OR stress management OR music therap* OR holistic need* assessment OR HNA OR reiki).ti,ab. | 662,624 | 698,882 |
| 22 | 20 OR 21 | 862,011 | 905,750 |
| 23 | 9 AND 14 AND 22 | 1,027 | 1,172 |
| 24 | (nutrition service or nutritional counseling or nutrition education or nutritional support or diet supplementation or dietary supplement or nutritional assessment or probiotic agent or synbiotic agent or dietary fiber or whey protein or plant protein or fortified food or vitamin supplementation or diet or diet therapy or antioxidant or omega 3 fatty acid).sh. | 806,821 | 841,842 |
| 25 | (Nutrition* OR diet* OR fiber OR fibre OR omega 3 OR omega3 OR antioxidant* OR malnutrition universal screening tool OR subjective global assessment OR SGA OR PG-SGA OR short nutrition assessment questionnaire OR SNAQ OR health package).ti,ab. | 1,760,553 | 1,836,746 |
| 26 | 24 OR 25 | 2,004,820 | 2,092,021 |
| 27 | 9 AND 14 AND 26 | 728 | 1,002 |
| 28 | limit 15 to (english language and yr="2024") | 11 | 4 |
| 29 | limit 19 to (english language and yr="2024") | 1,592 | 121 |
| 30 | limit 23 to (english language and yr="2024") | 968 | 77 |
| 31 | limit 27 to (english language and yr="2024") | 685 | 63 |

| S3 Table 6. Search strategy for AMED 15/10/24 | | | |
| --- | --- | --- | --- |
| Search no. | Search term | No. of results | New results |
| S1 | ti(cancer* OR neoplas* OR carcinoma* OR malignan* OR tumo*r* OR oncolog*) OR ab(cancer* OR neoplas* OR carcinoma* OR malignan* OR tumo*r* OR oncolog*) | 21,511 | 46 |
| S2 | SU.EXACT("NEOPLASMS") | 10,035 | 18 |
| S3 | S1 OR S2 | 23,368 | 46 |
| S4 | ti(Gyn*ecolog* OR ovar* OR uter* OR womb OR endometr* OR vagina* OR cervi* OR vulva* OR fallopian) OR ab(Gyn*ecolog* OR ovar* OR uter* OR womb OR endometr* OR vagina* OR cervi* OR vulva* OR fallopian | 6,572 | 3 |
| S5 | SU.EXACT("GENITAL DISEASES FEMALE") OR SU.EXACT("OVARIAN DIS") OR SU.EXACT("UTERINE CERVICAL DIS") OR SU.EXACT("UTERINE DIS") OR SU.EXACT("VAGINAL DIS") | 639 | 0 |
| S6 | S3 AND S4 | 990 | 2 |
| S7 | SU.EXACT("UTERINE CERVICAL NEOPLASMS") OR SU.EXACT("GENITAL NEOPLASMS FEMALE") OR SU.EXACT("OVARIAN NEOPLASMS") OR SU.EXACT("UTERINE NEOPLASMS") | 408 | 2 |
| S8 | S6 OR S7 | 1,055 | 2 |
| S9 | ti(Prehab* OR pre-hab* OR pre-rehab*) OR ab(Prehab* OR pre-hab* OR pre-rehab*) | 70 | 3 |
| S10 | S8 AND S9 | 3 | 0 |
| S11 | SU.EXACT("BRACHYTHERAPY") OR SU.EXACT("RADIOTHERAPY") | 748 | 1 |
| S12 | ti(Radiotherapy OR “Radiation therapy” OR EBRT OR Brachytherapy OR Chemoradiotherapy) OR ab(Radiotherapy OR “Radiation therapy” OR EBRT OR Brachytherapy OR Chemoradiotherapy) | 1,073 | 1 |
| S13 | S11 OR S12 | 1,331 | 2 |
| S14 | SU.EXACT("YOGA") OR SU.EXACT("RESISTANCE TRAINING") OR SU.EXACT("PILATES") OR SU.EXACT("MUSCLE STRETCHING EXERCISES") OR SU.EXACT("PHYSICAL FITNESS") OR SU.EXACT("RUNNING") OR SU.EXACT("EXERCISE TESTING") OR SU.EXACT("WEIGHT TRAINING") OR SU.EXACT("EXERCISE THERAPY") OR SU.EXACT("EXERCISE") OR SU.EXACT("SWIMMING") OR SU.EXACT("DANCING") OR SU.EXACT("EXERCISE TOLERANCE") OR SU.EXACT("BICYCLING") OR SU.EXACT("JOGGING") OR SU.EXACT("WALKING") | 35,116 | 63 |
| S15 | ti(Exercise OR “aerobics” OR dancing OR dance OR run* OR jog* OR walk* OR “high-intensity interval” OR “high intensity interval” OR "HIIT" OR “Low-intensity steady state” OR “Low intensity steady state” OR "LISS" OR “physical activity” OR endurance OR training OR conditioning OR fitness OR weightlifting OR “weight lifting” OR cal*isthenics OR plyometric OR kegel OR “pelvic floor” OR dilators OR pilates OR yoga OR stretch* OR flexibility OR swimming OR cycling) OR ab(Exercise OR “aerobics” OR dancing OR dance OR run* OR jog* OR walk* OR “high-intensity interval” OR “high intensity interval” OR "HIIT" OR “Low-intensity steady state” OR “Low intensity steady state” OR "LISS" OR “physical activity” OR endurance OR training OR conditioning OR fitness OR weightlifting OR “weight lifting” OR cal*isthenics OR plyometric OR kegel OR “pelvic floor” OR dilators OR pilates OR yoga OR stretch* OR flexibility OR swimming OR cycling) | 54,071 | 179 |
| S16 | S14 OR S15 | 60,999 | 180 |
| S17 | S8 AND S13 AND S16 | 2 | 0 |
| S18 | SU.EXACT("RELAXATION") OR SU.EXACT("PSYCHIATRY") OR SU.EXACT("VISUALIZATION") OR SU.EXACT("STRESS PSYCHOLOGICAL") OR SU.EXACT("SEX COUNSELING") OR SU.EXACT("PSYCHOLOGY SOCIAL") OR SU.EXACT("PSYCHOLOGY") OR SU.EXACT("MUSIC THERAPY") OR SU.EXACT("COGNITIVE THERAPY") OR SU.EXACT("MEDITATION") OR SU.EXACT("HOLISTIC HEALTH") OR SU.EXACT("HOLISTIC NURSING") OR SU.EXACT("IMAGERY") OR SU.EXACT("MIND BODY MEDICINE") OR SU.EXACT("SUGGESTION") OR SU.EXACT("BEHAVIOR THERAPY") OR SU.EXACT("HYPNOSIS") OR SU.EXACT("DESENSITIZATION") OR SU.EXACT("PSYCHOTHERAPY") OR SU.EXACT("COUNSELING") | 52,400 | 125 |
| S19 | ti("Psycho* intervention" OR "Psycho* interventions" OR "psycho* support" OR "well-being" OR "wellbeing" OR psychotherap* OR counsel*ing OR "support group" OR "support groups" OR "cognitive therap*" OR "cognitive behavio*r* therapy" OR CBT OR "dialectical behavio*r therapy" OR "cognitive restructuring" OR "eye movement desensitisation and reprogramming" OR “eye movement desensitization and reprogramming” OR mindfulness OR relaxation OR meditation OR hypnotherap* OR "stress management" OR "music therap*" OR "holistic need* assessment" OR HNA OR reiki) OR ab("Psycho* intervention" OR "Psycho* interventions" OR "psycho* support" OR "well-being" OR "wellbeing" OR psychotherap* OR counsel*ing OR "support group" OR "support groups" OR "cognitive therap*" OR "cognitive behavio*r* therapy" OR CBT OR "dialectical behavio*r therapy" OR "cognitive restructuring" OR "eye movement desensitisation and reprogramming" OR “eye movement desensitization and reprogramming” OR mindfulness OR relaxation OR meditation OR hypnotherap* OR "stress management" OR "music therap*" OR "holistic need* assessment" OR HNA OR reiki) | 39,511 | 46 |
| S20 | S18 OR S19 | 80,933 | 136 |
| S21 | S8 AND S13 AND S20 | 16 | 0 |
| S22 | SU.EXACT("PROBIOTICS") OR SU.EXACT("NUTRITION") OR SU.EXACT("DIET THERAPY") OR SU.EXACT("ANTIOXIDANTS") OR SU.EXACT("DIETARY SUPPLEMENTS") OR SU.EXACT("NUTRITIONAL STATUS") OR SU.EXACT("DIETARY PROTEINS") OR SU.EXACT("DIETARY FIBER") | 8,874 | 7 |
| S23 | ti(Nutrition* OR diet* OR fiber OR fibre OR “omega-3” OR “omega 3” OR “omega3” OR antioxidant* OR “malnutrition universal screening tool” OR “MUST” OR “subjective global assessment” OR SGA OR PG-SGA OR “short nutrition assessment questionnaire” OR SNAQ OR “health package”) OR ab(Nutrition* OR diet* OR fiber OR fibre OR “omega-3” OR “omega 3” OR “omega3” OR antioxidant* OR “malnutrition universal screening tool” OR “MUST” OR “subjective global assessment” OR SGA OR PG-SGA OR “short nutrition assessment questionnaire” OR SNAQ OR “health package”) | 12,978 | 25 |
| S24 | S22 OR S23 | 16,334 | 26 |
| S25 | S8 AND S13 AND S24 | 4 | 0 |

| S3 Table 7. Search strategy for BNI 14/10/2024 | | | |
| --- | --- | --- | --- |
| Search no. | Search term | No. of results | New results |
| 1 | tiab(cancer OR neoplas* OR carcinoma OR malignan* OR tumo?r OR oncolog*) | 65,691 | 1,786 |
| 2 | MAINSUBJECT.EXACT(“Tumors”) | 4,074 | 104 |
| 3 | [S1] OR [S2] | 66,908 | 1,800 |
| 4 | tiab(Gyn?ecolog* OR ovar* OR uter* OR womb OR endometr* OR vagina* OR cervi* OR vulva* OR fallopian) | 16,490 | 349 |
| 5 | MAINSUBJECT.EXACT("Gynecology") | 3,966 | 82 |
| 6 | [S4] OR [S5] | 18,482 | 388 |
| 7 | [S3] AND [S6] | 5,986 | 132 |
| 8 | MAINSUBJECT.EXACT("Ovarian cancer") OR MAINSUBJECT.EXACT("Uterine cancer") OR MAINSUBJECT.EXACT("Genital cancers") OR MAINSUBJECT.EXACT("Endometrial cancer") OR MAINSUBJECT.EXACT("Cervical cancer") | 4,887 | 105 |
| 9 | [S7] OR [S8] | 7,506 | 160 |
| 10 | tiab(Prehabilitation OR Prehab OR pre-habilitation OR pre-hab OR pre-rehab OR pre-rehabilitation) | 54 | 5 |
| 11 | [S9] AND [S10] | 2 | 0 |
| 12 | MAINSUBJECT.EXACT("Radiation therapy") | 2,907 | 106 |
| 13 | tiab(Radiotherapy OR “Radiation therapy” OR EBRT OR Brachytherapy OR Chemoradiotherapy) | 2,829 | 76 |
| 14 | [S12] OR [S13] | 4,131 | 112 |
| 15 | [S11] AND [S14] | 0 | 0 |
| 16 | MAINSUBJECT.EXACT("Exercise") OR MAINSUBJECT.EXACT("Aerobics") OR MAINSUBJECT.EXACT("Running") OR MAINSUBJECT.EXACT("Walking") OR MAINSUBJECT.EXACT("Interval training") OR MAINSUBJECT.EXACT("Physical fitness") OR MAINSUBJECT.EXACT("Fitness training programs") OR MAINSUBJECT.EXACT("Cardiac stress tests") OR MAINSUBJECT.EXACT("Physical fitness tests") OR MAINSUBJECT.EXACT("Weightlifting") OR MAINSUBJECT.EXACT("Strength training") OR MAINSUBJECT.EXACT("Isometric exercise") OR MAINSUBJECT.EXACT("Pilates") OR MAINSUBJECT.EXACT("Yoga") OR MAINSUBJECT.EXACT("Stretching") OR MAINSUBJECT.EXACT("Swimming") OR MAINSUBJECT.EXACT("Bicycling") | 13,772 | 298 |
| 17 | tiab(Exercise OR aerobics OR dance OR dancing OR run* OR jog* OR sprint* OR walk* OR “high intensity interval” OR HIIT OR “Low intensity steady state” OR LISS OR “physical activity” OR endurance OR training OR fitness OR weightlifting OR “weight lifting” OR cal?isthenics OR plyometric OR kegel OR “pelvic floor” OR dilator* OR pilates OR yoga OR stretch* OR flexibility OR swimming OR cycling) | 68,051 | 1,737 |
| 18 | [S16] OR [S17] | 71,728 | 1,819 |
| 19 | [S9] AND [S14] AND [S18] | 29 | 2 |
| 20 | MAINSUBJECT.EXACT("Psychiatric-mental health nursing") OR MAINSUBJECT.EXACT("Psychotherapy") OR MAINSUBJECT.EXACT("Group therapy") OR MAINSUBJECT.EXACT("Support groups") OR MAINSUBJECT.EXACT("Counseling") OR MAINSUBJECT.EXACT("Counseling psychology") OR MAINSUBJECT.EXACT("Cognitive therapy") OR MAINSUBJECT.EXACT("Desensitization (Psychology)") OR MAINSUBJECT.EXACT("Mind body relationship") OR MAINSUBJECT.EXACT("Mindfulness") OR MAINSUBJECT.EXACT("Relaxation therapy") OR MAINSUBJECT.EXACT("Visualization") OR MAINSUBJECT.EXACT("Meditation") OR MAINSUBJECT.EXACT("Hypnosis") OR MAINSUBJECT.EXACT("Hypnotherapy") OR MAINSUBJECT.EXACT("Music therapy") OR MAINSUBJECT.EXACT("Holistic medicine") OR MAINSUBJECT.EXACT("Holistic nursing") OR MAINSUBJECT.EXACT("Reiki") | 29,335 | 416 |
| 21 | tiab(“{Psychological intervention}” OR “{Psychosocial intervention}” OR “psychological support” OR “psychosocial support” OR wellbeing OR “well-being” OR psychotherap* OR counsel?ing OR “{support group}” OR “{cognitive therapy}” OR “{cognitive behavior therapy}” OR CBT OR “{dialectical behavior therapy}” OR “cognitive restructuring” OR “{eye movement desensitization and reprogramming}” OR EMDR OR mindfulness OR relaxation OR meditation OR hypnotherap* OR “stress management” OR “{music therapy}” OR “{holistic need assessment}” OR HNA OR reiki) | 34,828 | 949 |
| 22 | [S20] OR [S21] | 56,295 | 1,197 |
| 23 | [S9] AND [S14] AND [S22] | 20 | 1 |
| 24 | MAINSUBJECT.EXACT("Nutrition") OR MAINSUBJECT.EXACT("Nutrition research") OR MAINSUBJECT.EXACT("Nutrition therapy") OR MAINSUBJECT.EXACT("Nutrition education") OR MAINSUBJECT.EXACT("Diet") OR MAINSUBJECT.EXACT("Dietary supplements") OR MAINSUBJECT.EXACT("Probiotics") OR MAINSUBJECT.EXACT("Prebiotics") OR MAINSUBJECT.EXACT("Dietary fiber") OR MAINSUBJECT.EXACT("Food fortification") OR MAINSUBJECT.EXACT("Antioxidants") | 21,361 | 390 |
| 25 | tiab(Nutrition* OR diet* OR fiber OR fibre OR “omega 3” OR “omega3” OR antioxidant* OR “malnutrition universal screening tool” OR “MUST” OR “subjective global assessment” OR SGA OR “PG SGA” OR “short nutrition assessment questionnaire” OR SNAQ OR “health package”) | 50,074 | 1,173 |
| 26 | [S24] OR [S25] | 59,119 | 1,252 |
| 27 | [S9] AND [S14] AND [S26] | 16 | 2 |
| 28 | [S19] OR [S23] OR [S27] | 52 | 5 |

| S3 Table 8. Search strategy for Cochrane 14/10/2024 | | | |
| --- | --- | --- | --- |
| Search no. | Search term | No. of results | New results |
| S1 | (cancer* OR neoplas* OR carcinoma* OR malignan* OR tumo?r* OR oncolog*):ti OR (cancer* OR neoplas* OR carcinoma* OR malignan* OR tumo?r* OR oncolog*):ab | 248,866 | 13,725 |
| S2 | [mh ^Neoplasms] | 10,242 | 288 |
| S3 | #1 OR #2 | 249,861 | 13,736 |
| S4 | (Gyn?ecolog* OR ovar* OR uter* OR womb OR endometr* OR vagina* OR cervi* OR vulva* OR fallopian):ti OR (Gyn?ecolog* OR ovar* OR uter* OR womb OR endometr* OR vagina* OR cervi* OR vulva* OR fallopian):ab | 92,456 | 5,086 |
| S5 | [mh ^Gynecology] | 266 | 6 |
| S6 | [mh ^"Genital Diseases, Female"] OR [mh ^"Ovarian Diseases"] OR [mh ^"Uterine Diseases"] OR [mh ^"Uterine Cervical Diseases"] OR [mh ^"Vulvar Diseases"] OR [mh ^"Vaginal Diseases"] OR [mh ^"Adnexal Diseases"] OR [mh ^"Fallopian Tube Diseases"] | 1,756 | 39 |
| S7 | #4 OR #5 OR #6 | 92,881 | 5,089 |
| S8 | #3 AND #7 | 22,727 | 1,209 |
| S9 | [mh ^"Genital Neoplasms, Female"] OR [mh ^"Ovarian Neoplasms"] OR [mh ^"Carcinoma, Ovarian Epithelial"] OR [mh ^"Uterine Neoplasms"] OR [mh ^"Uterine Cervical Neoplasms"] OR [mh ^"Endometrial Neoplasms"] OR [mh ^"Vaginal Neoplasms"] OR [mh ^"Vulvar Neoplasms"] OR [mh ^"Fallopian Tube Neoplasms"] | 9,173 | 228 |
| S10 | #8 OR #9 | 24,194 | 1,227 |
| S11 | (Prehab* OR pre NEXT hab* OR pre NEXT rehab*):ti OR (Prehab* OR pre NEXT hab* OR pre NEXT rehab*):ab | 781 | 133 |
| S12 | #10 AND #11 | 23 | 7 |
| S13 | [mh ^Radiotherapy] OR [mh ^Brachytherapy] OR [mh ^Chemoradiotherapy] OR [mh ^"Chemoradiotherapy, Adjuvant"] OR [mh ^"Radiotherapy, Conformal"] | 4,942 | 81 |
| S14 | (Radiotherapy OR “Radiation therapy” OR EBRT OR Brachytherapy OR Chemoradiotherapy):ti OR (Radiotherapy OR “Radiation therapy” OR EBRT OR Brachytherapy OR Chemoradiotherapy):ab | 38,425 | 1,945 |
| S15 | #13 OR #14 | 39,201 | 1,952 |
| S16 | #12 AND #15 | 2 | 0 |
| S17 | [mh ^Exercise] OR [mh ^"Exercise Therapy"] OR [mh ^Dancing] OR [mh ^"Dance Therapy"] OR [mh ^"Aquatic Therapy"] OR [mh ^Running] OR [mh ^Jogging] OR [mh ^Walking] OR [mh ^"High-Intensity Interval Training"] OR [mh ^"Circuit-Based Exercise"] OR [mh ^"Endurance Training"] OR [mh ^"Exercise Test"] OR [mh ^"Walk Test"] OR [mh ^"Exercise Tolerance"] OR [mh ^"Weight Lifting"] OR [mh ^"Resistance Training"] OR [mh ^"Plyometric Exercise"] OR [mh ^"Exercise Movement Techniques"] OR [mh ^Yoga] OR [mh ^"Muscle Stretching Exercises"] OR [mh ^Swimming] OR [mh ^Bicycling] | 57,539 | 1,525 |
| S18 | (Exercise OR “aerobics” OR dancing OR dance OR run* OR jog* OR walk* OR “high intensity interval” OR HIIT OR “Low intensity steady state” OR LISS OR “physical activity” OR endurance OR training OR fitness OR weightlifting OR “weight lifting” OR cal?isthenics OR plyometric OR kegel OR “pelvic floor” OR dilator* OR pilates OR yoga OR stretch* OR flexibility OR swimming OR cycling):ti OR (Exercise OR “aerobics” OR dancing OR dance OR run* OR jog* OR walk* OR “high intensity interval” OR HIIT OR “Low intensity steady state” OR LISS OR “physical activity” OR endurance OR training OR fitness OR weightlifting OR “weight lifting” OR cal?isthenics OR plyometric OR kegel OR “pelvic floor” OR dilator* OR pilates OR yoga OR stretch* OR flexibility OR swimming OR cycling):ab | 268,191 | 19,932 |
| S19 | #17 OR #18 | 272,478 | 19,973 |
| S20 | #10 AND #15 AND #19 | 206 | 24 |
| S21 | [mh ^"Psychosocial Intervention"] OR [mh ^"Psychosocial Support Systems"] OR [mh ^"Psychiatric Rehabilitation"] OR [mh ^"Psychological Well-Being"] OR [mh ^Psychotherapy] OR [mh ^"Psychotherapy, Brief"] OR [mh ^"Schema Therapy"] OR [mh ^"Psychotherapy, Group"] OR [mh ^Counseling] OR [mh ^"Couples Therapy"] OR [mh ^"Sex Counseling"] OR [mh ^"Cognitive Behavioral Therapy"] OR [mh ^"Dialectical Behavior Therapy"] OR [mh ^"Cognitive Restructuring"] OR [mh ^"Desensitization, Psychologic"] OR [mh ^"Eye Movement Desensitization Reprocessing"] OR [mh ^"Virtual Reality Exposure Therapy"] OR [mh ^"Mind-Body Therapies"] OR [mh ^Mindfulness] OR [mh ^" Relaxation Therapy"] OR [mh ^"Relaxation"] OR [mh ^"Imagery, Psychotherapy"] OR [mh ^Meditation] OR [mh ^Hypnosis] OR [mh ^"Music Therapy"] OR [mh ^"Holistic Health"] OR [mh ^"Therapeutic Touch"] | 28,728 | 779 |
| S22 | (Psych* NEXT intervention* OR “psychological support” OR “psychosocial support” OR “well being” OR “wellbeing” OR psychotherap* OR counsel?ing OR “support group” OR “cognitive therapy” OR “cognitive therapies” OR “cognitive behavior therapy” OR “cognitive behaviour therapy” OR “cognitive behavioral therapy” OR “cognitive behavioural therapy” OR CBT OR “dialectical behavior therapy” OR “dialectical behaviour therapy” OR “cognitive restructuring” OR “eye movement desensitisation and reprogramming” OR “eye movement desensitization and reprogramming” OR EMDR OR mindfulness OR relaxation OR meditation OR hypnotherapy OR “stress management” OR “music therapy” OR “holistic need assessment” OR “holistic needs assessment” OR HNA OR reiki):ti OR (Psych* NEXT intervention* OR “psychological support” OR “psychosocial support” OR “well being” OR “wellbeing” OR psychotherap* OR counsel?ing OR “support group” OR “cognitive therapy” OR “cognitive therapies” OR “cognitive behavior therapy” OR “cognitive behaviour therapy” OR “cognitive behavioral therapy” OR “cognitive behavioural therapy” OR CBT OR “dialectical behavior therapy” OR “dialectical behaviour therapy” OR “cognitive restructuring” OR “eye movement desensitisation and reprogramming” OR “eye movement desensitization and reprogramming” OR EMDR OR mindfulness OR relaxation OR meditation OR hypnotherapy OR “stress management” OR “music therapy” OR “holistic need assessment” OR “holistic needs assessment” OR HNA OR reiki):ab | 99,196 | 7,892 |
| S23 | #21 OR #22 | 108,561 | 8,082 |
| S24 | #10 AND #15 AND #23 | 98 | 10 |
| S25 | [mh ^"Nutrition Therapy"] OR [mh ^"Diet Therapy"] OR [mh ^"Nutritional Support"] OR [mh ^"Dietary Supplements"] OR [mh ^"Dietary Fiber"] OR [mh ^Probiotics] OR [mh ^Prebiotics] OR [mh ^Synbiotics] OR [mh ^"Whey Proteins"] OR [mh ^"Plant Proteins, Dietary"] OR [mh ^"Food, Fortified"] OR [mh ^Antioxidants] OR [mh ^"Nutrition Assessment"] | 29,686 | 694 |
| S26 | (Nutrition* OR diet* OR fiber OR fibre OR “omega 3” OR “omega3” OR antioxidant* OR “malnutrition universal screening tool” OR “MUST” OR “subjective global assessment” OR SGA OR “PG SGA” OR “short nutrition assessment questionnaire” OR SNAQ OR “health package”):ti OR (Nutrition* OR diet* OR fiber OR fibre OR “omega 3” OR “omega3” OR antioxidant* OR “malnutrition universal screening tool” OR “MUST” OR “subjective global assessment” OR SGA OR “PG SGA” OR “short nutrition assessment questionnaire” OR SNAQ OR “health package”):ab | 174,056 | 12,409 |
| S27 | #25 OR #26 | 187,035 | 12,756 |
| S28 | #10 AND #15 AND #27 | 285 | 26 |
| S29 | #16 OR #20 OR #24 OR #28 | 507 | 48 |
